# Visualizing the orientational dependence of an intermolecular potential

**DOI:** 10.1038/ncomms10621

**Published:** 2016-02-16

**Authors:** Adam Sweetman, Mohammad A. Rashid, Samuel P. Jarvis, Janette L. Dunn, Philipp Rahe, Philip Moriarty

**Affiliations:** 1 School of Physics and Astronomy, University of Nottingham, Nottingham NG7 2RD, UK

## Abstract

Scanning probe microscopy can now be used to map the properties of single molecules with intramolecular precision by functionalization of the apex of the scanning probe tip with a single atom or molecule. Here we report on the mapping of the three-dimensional potential between fullerene (C_60_) molecules in different relative orientations, with sub-Angstrom resolution, using dynamic force microscopy (DFM). We introduce a visualization method which is capable of directly imaging the variation in equilibrium binding energy of different molecular orientations. We model the interaction using both a simple approach based around analytical Lennard–Jones potentials, and with dispersion-force-corrected density functional theory (DFT), and show that the positional variation in the binding energy between the molecules is dominated by the onset of repulsive interactions. Our modelling suggests that variations in the dispersion interaction are masked by repulsive interactions even at displacements significantly larger than the equilibrium intermolecular separation.

## Abstract (web summary)

Intermolecular interactions underpin an array of physical and chemical phenomena. Here, the authors probe the three dimensional potential between fullerene molecules in different orientations showing that the positional variation in the intermolecular binding energy is dominated repulsive interactions.

## Graphical abstract

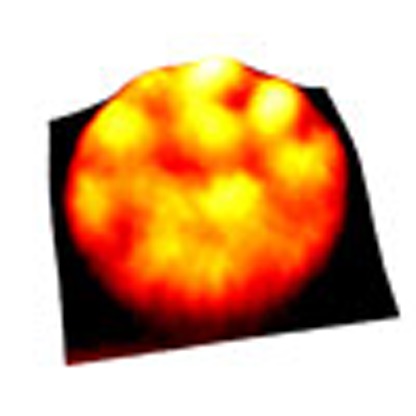

The nature of intermolecular interactions underpins a vast array of physical and chemical phenomena, and is a scientific theme that straddles the disciplines of physics, chemistry and biology. Particular impetus has been given to the study of intermolecular forces at the single-molecule level due to the stunning advances in ultrahigh resolution scanning probe imaging pioneered by Gross *et al*.^1^. Three-dimensional (3D) force maps were acquired over planar organic molecules that bore a striking resemblance to the classic textbook ‘ball-and-stick' models. These advances were first realized via the controllable functionalization of the scanning probe tip with a single pre-selected atom or molecule, which provides a unique level of control with which to investigate the atomic and molecular scale properties of matter, and also helps to eliminate the most troublesome aspect of scanning probe experiments, that is, the uncertainty surrounding the tip structure.

Although this tip functionalization strategy is now commonly applied to single CO molecules to allow intramolecular imaging^1^,^2^, the technique has application well beyond imaging, and similar protocols have also been used to study the electronic^3^,^4^ and mechanical^5^ properties of single molecules trapped in the tip-sample junction, and to quantitatively measure intermolecular interactions^6^,^7^,^8^. There has also been considerable interest centred around the possibility of using this technique to directly visualize intermolecular interactions^9^, although considerable debate surrounds the interpretation of these results^2^,^10^,^11^,^12^.

In this paper, we discuss the results of a series of experiments—and their interpretation on the basis of both simple analytical potentials and DFT—that map the orientational dependence of the 3D potential between two-complex molecules. By measuring the full 3D potential we are able to apply a novel visualization method that directly shows the variation in the equilibrium binding energy for the molecular system for different relative orientations of the molecules. We also discuss the feasibility of detecting the variation in dispersion forces due to molecular rotation via DFM.

## Results

### Experimental results

**Figure 1:**
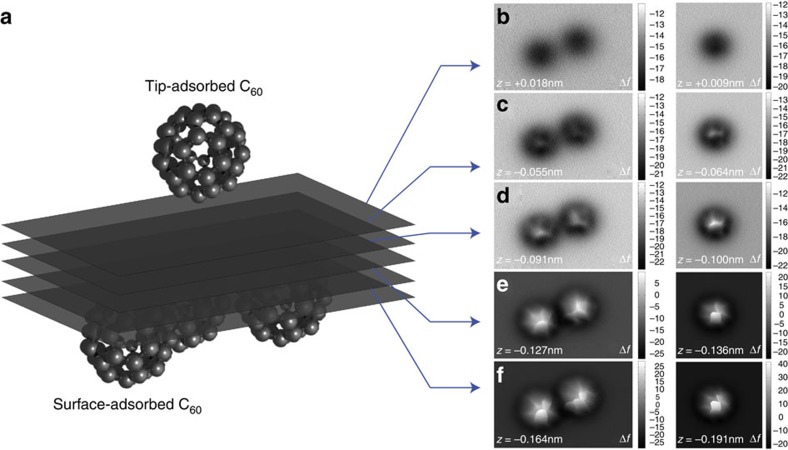
Experimental data acquisition protocol. (**a**) cartoon showing the method of data acquisition for 3D potential mapping—a single C_60_ molecule is attached to the tip of the scanning probe microscope and brought close to a group of surface-adsorbed molecules. Constant height scans are acquired at decreasing tip-sample separation, with active drift compensation between each scan, and the variation in the frequency shift Δ*f* measured. (**b**–**f**) representative Δ*f* images (in Hz) at decreasing tip-sample height. Tip-sample heights shown for each image are given relative to the Δ*f* set point used for atom-tracking over the molecule. The slightly different *z* heights for the two data sets result from the slightly different tracking heights used in each case. Image sizes: 3.5 × 2.2 nm^2^ and 2.5 × 2.5 nm^2^.

Figure 1 shows representative constant height Δ*f* images, taken from a 3D grid, acquired at decreasing tip-sample separation over three surface-adsorbed C_60_ molecules in different orientations, using a C_60_-terminated tip.

At larger separations a featureless circular attractive interaction is observed (Fig. 1b), but on closer approach intricate intramolecular features are resolved (Fig. 1c,d), followed by their intense ‘sharpening' (Fig. 1e,f). This evolution in contrast is similar to the onset of sub-molecular features during imaging of planar molecules with flexible tips^1^,^12^. However, because in this experiment both molecules have a complex structure, the intramolecular features in these images cannot be easily assigned to the molecular structure of the surface molecule as is the case for images taken with simple (that is, atomic point-like) tip terminations.

**Figure 2:**
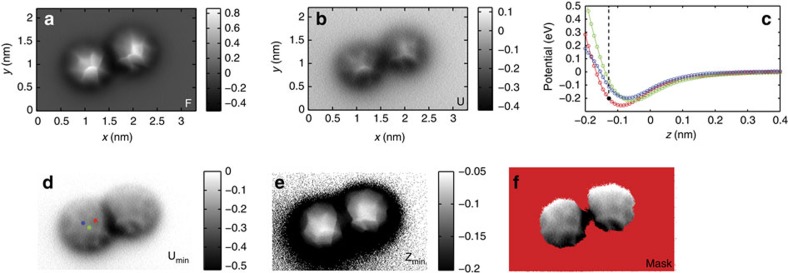
Experimental measurement of variation in potential between C_60_ molecules in different orientations. Constant height images of (**a**) force (in nN) and (**b**) energy (in eV). (**c**) Representative *U*(*z*) curves taken at different positions across the left hand C_60_ molecule, dotted line shows the height of the force and energy slices shown in **a**,**b**. (**d**) Image showing the variation in the value of the energy at the minimum in the *U*(*z*) curve (in eV) at each position in the grid. The positions of the curves shown in **c** are marked. (**e**) As for **d** but showing instead the *z* height at which the minimum occurs, note that the black regions indicate parts of the grid where the minimum is found at the lowest tip-sample separation (that is, no turnaround detected). (**f**) Variation in energy minimum masked using the minimum in *z* position. Red shading indicates locations where the minimum in the intermolecular potential is not present in the *U*(*z*) curve.

Converting the acquired Δ*f* grid into a map of potential allows us to create similar constant height images of the tip-sample force and potential (Fig. 2a,b and Supplementary Fig. 1). Although constant height slices of force and energy provide the closest visual analogue to how the data are collected, these images necessarily conflate the value of the tip-sample energy and the topographic height of the molecule at a given position. Consequently, topographically higher features dominate the constant height image due to their being effectively shifted in *z*, even if the range of energies at these positions is identical to other locations over the molecule.

Representative single *U*(*z*) curves may be extracted (Fig. 2c) and allow a selection of the energy minimum values at different positions to be observed, but this is an indirect, and not necessarily intuitive, method of analysing the variation in intermolecular potential across the molecules.

In Fig. 2d, we instead show an image constructed by searching each vertical column in the 3D data set (that is, each *U*(*z*) curve) for the value of the potential energy minimum, and then projecting this minimum value over the *xy* plane of the grid, which we hereafter refer to as a ‘*U*_*min*_' image. This provides an immediate and intuitive way of visualizing the strength of the equilibrium interaction as the relative position of the tip- and sample-adsorbed molecules is varied. We note that, because of the near-unique high-rotational symmetry of the C_60_ molecules, displacements in the *xy* plane should be equivalent to changes in rotational orientation.

We highlight here that some care must be taken in the interpretation of these images, as the value of the minimum in the potential energy curve only has a directly interpretable physical meaning when the actual minimum of the potential is present in a given *U*(*z*) curve (that is, the turn-around in the *U*(*z*) curve is present in the data set). If the minimum is not reached, then the closest point of approach will usually be identified as the minimum value. We therefore also map the height of the potential energy minimum in terms of *z*, which yields a complementary map of *z*_*min*_. By masking the *U*_*min*_ map with the *z*_*min*_ map we can exclude those curves which do not contain the *U*(*z*) turn-around, and visualize only the region of the image, which can be interpreted directly as representing the intermolecular interaction minimum (Fig. 2f). Application of this visualization technique also reveals a gradient in the value of the minimum in the potential across the molecule, most likely related to an asymmetric mounting of the molecule on the tip. Since this gradient directly affects the spread in the energy values we therefore only discuss the variation observed in the region located over the centre of the molecule, where the variation due to the gradient is small compared to the variation produced by the changes in molecular orientation (Supplementary Figs 16–18).

The same technique may also be applied to the 3D force field and raw 3D Δ*f* measurements (see Supplementary Fig. 3). Although these maps do not have such a direct physical interpretation as for the minimum in the potential, they still provide an extremely powerful technique for visualizing the relative interaction over the molecule. Interestingly, we note a strong qualitative similarity in the appearance of these images and recent data acquired using a profile-corrected constant height technique by Moreno *et al*.^13^. We also note that Mohn *et al*.^14^ recovered a pssuedo-topographic Δ*f* image from a 3D data set, and experimentally it has been shown how to operate in the Δ*f*=0 regime^15^, which might, in principle, produce similar imaging if applied to intermolecular measurements. Critically, however, none of these earlier works directly measured and visualised the physical quantity of interest here: the variation in the value of the minimum in the intermolecular potential.

Our data demonstrate that as the relative orientations of the tip and surface C_60_ molecules are varied the potential minimum between the two molecules varies of the order 60 meV. A key question is therefore—what is the origin of this variation? A common approach to evaluating the C_60_–C_60_ intermolecular interaction is to model molecular energy variation using the Girifalco potential^7^,^16^, but this simplified model assumes a uniform spherical interaction, and does not give any information about sub-molecular variation in the potential. In particular, given the extended 3D nature of the molecule, it is not immediately clear how the attractive and repulsive components of the intermolecular potential contribute to the variation in the magnitude, and position, of the energy minimum. Following recent studies investigating the variation in dispersion force as a function of molecular size^6^, there is also an open question as to whether the difference in the dispersion interaction can be observed for changes in the orientation of extended molecules. C_60_, with its near-spherical symmetry represents a particularly important test bed for this hypothesis.

### Computational results

**Figure 3:**
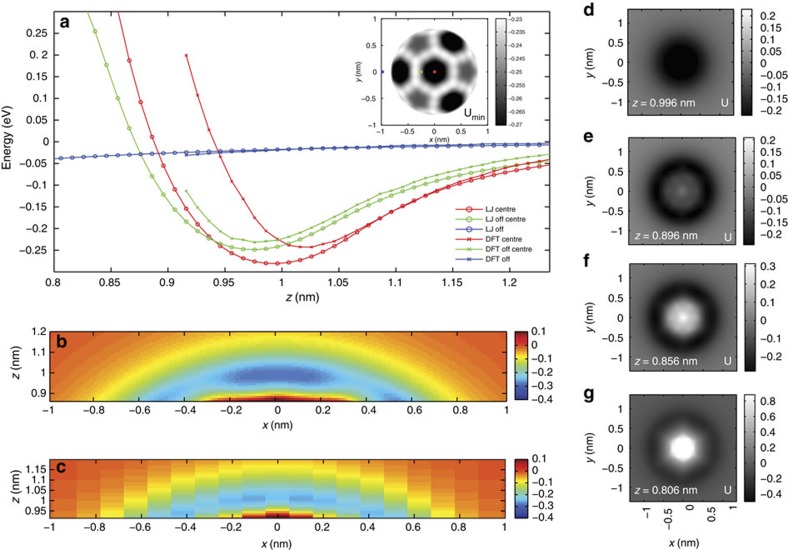
Modelling the C_60_–C_60_ interaction. (**a**) Comparison of potentials calculated by L–J modelling and DFT. *z* heights are defined as the vertical separation of the two molecules measured from the molecular centres. Curves are shown for the same initial *xy* coordinates in both simulations. Inset: Complete *xy U*_*min*_ (in eV) image for L-J simulation. (**b**,**c**) *xz* plot of calculated energies for L-J and DFT simulations, respectively. (**d**–**g**) Constant height *xy* energy images (in eV) from L–J simulations, relative heights labelled on each image.

To interpret our results, we modelled our experimental system with two different approaches (as described in the Methods section). First, we used a simple Lennard–Jones (L–J) potential for two C_60_ molecules, coupled with a modified version of the flexible tip model introduced by Hapala *et al*.^12^, to simulate the C_60_ interaction. The simple nature of the model means that it is computationally inexpensive and thus can be exploited to generate high-resolution 3D data sets of comparable data density to those we obtain experimentally (Fig. 3d–g). Second, to test the validity of our empirical model, we compare the results of the L-J calculations to simulations of the same C_60_–C_60_ interaction performed using the *ab initio* CP2K DFT code. The significant computational cost of the *ab initio* simulations precludes the calculation of a full 3D grid as for the L–J simulations, and we therefore instead compare 2D *xz* slices taken across the centre of the molecule–molecule interaction (Fig. 3b,c). In this comparison, we modelled a prototypical high symmetry orientation (hexagon face on hexagon face, hereafter referred to as Hex–Hex) for the two molecules using both simulations methods. In general, we find good agreement between the two techniques, noting in particular that the potential gradients in both the attractive and repulsive branches of the potential curve are very similar. We do, however, observe some quantitative differences between the empirical L–J simulations and *ab initio* DFT. Specifically, for the L–J parameters chosen (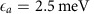 and *r*_*a*_=1.966 Å (ref. 16)), the maximum depth and width of the well is slightly larger that for the DFT simulations, as is the variation in the range of U_*min*_ (Δ*U*_*min*_ for L–J *xz* plot ∼50 meV compared with ∼20 meV for the DFT *xz* plot over the same range (Supplementary Figs 20–22)). We note, however, that variation in the positions of the minimum is almost identical (Δ*Z*_*min*_ for L–J *xz* plot ∼0.09 nm, compared with Δ*Z*_*min*_∼0.11 nm for the DFT *xz* plot). Furthermore, it is clear that tuning the choice of L–J parameters based on the DFT results could improve the quantitative agreement between the two simulation methods, but here we prefer to use those L–J parameters derived from previous experimental work and which are also consistent with our earlier publications, rather than arbitrarily adjusting the L–J parameters. These results imply that while the L–J model is a simplification of the complex intermolecular interaction, it nonetheless appears to be sufficient to model much of the essential physics underpinning the variation in intermolecular potential.

Although we stress that the high-symmetry Hex–Hex configuration used in the simulations is not the configuration of the C_60_ molecules in the experimental data set shown in Fig. 2, we nonetheless observe a number of qualitatively similar features in both the simulations and experiment. In particular, the simulations reproduce the ‘sharpening' of the features observed in the constant-height experimental images, in line with the sharpening reported for CO-terminated tips. In addition, the appearance of the simulated U_*min*_ image is qualitatively similar to that acquired in the experiment, which reveals the complex variation in potential minimum as the molecular positions are varied. Interestingly, the L–J simulations overestimate the depth of the potential relative to the DFT calculations, but better reproduce the variation in *U*_*min*_ observed experimentally, with a variation of ∼50 to 60 meV in the *U*_*min*_ image depending on molecular orientation (Supplementary Figs 16–18). We also note that simulations performed with other tip-sample molecular configurations, such as those found for C_60_ adsorption on the Si(111)-7 × 7 substrate, produce much more complex patterns in the constant height, and *U*_*min*_, images (see Supplementary Figs 7–13), qualitatively similar to those observed experimentally.

## Discussion

**Figure 4:**
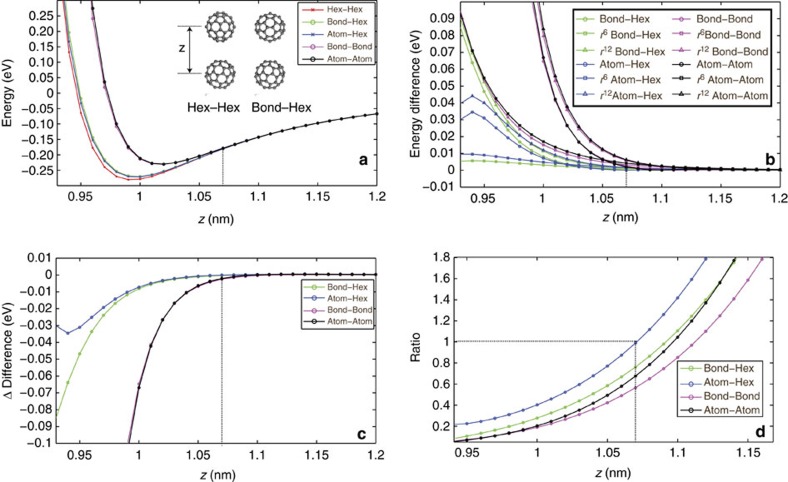
Investigating the origin of the variation in potential energy minimum. (**a**) Variation in potential energy between two C_60_ molecules due to variation in rotational orientation, the terms in the legend refer to the facing part of the molecule. Inset, representative ball-and-stick models showing two of the simulated orientations. (**b**) Modulus of the difference in *U*_total_, 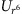, and 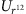, between stated orientation and the Hex–Hex orientation. (**c**) Difference of variation in 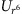 and 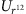 terms (that is, 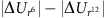). Negative values indicate the 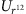 term dominates the difference in energy between the two configurations. (**d**) Ratio of variation in 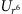 and 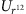 terms. Values above 1 indicate the 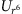 term has a larger contribution. Dotted lines indicate the separation at which the 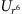 term first becomes dominant.

Because of the simple additive, and analytical, nature of the L–J model, it is possible to decompose the interaction into its attractive and repulsive components, and ascertain if we might in principle be able to observe rotational variation in the dispersion interaction between the C_60_ molecules. To assess the relative influence of the repulsive and attractive elements of the potential on the value of the potential minimum we investigated the change in the potential for several orientations of the tip and sample C_60_ (Fig. 4a). We then plot the modulus (i.e. the absolute value) of the differences in the total energies, and the separate energies from the *r*^6^ and *r*^12^ terms, between these orientations and the high symmetry ‘Hex–Hex' configuration (Fig. 4b), and then extract the differences in these 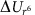 and 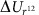 terms (Fig. 4c). Specifically, we define 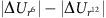 as the modulus of the difference of the 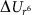 term (between the stated orientation and the Hex–Hex orientation), minus the modulus of the difference in the 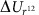 terms (between the stated orientation and the Hex–Hex orientation).

This quantity gives the relative influence of the two terms in defining the difference in the energy curves between the two orientations. If the difference in *r*^6^ terms at a given separation is greater (that is, dispersion forces vary significantly between different orientations), then this quantity will be positive. If the difference in *r*^12^ terms is large (that is, Pauli forces vary significantly between different orientations), then it will be negative. If both quantities contribute equally to the difference in energy, then the term will be approximately zero. Surprisingly, we observe that the difference is negative and, consequently, the differences observed in the total energies, even in the part of the well where the potential gradient is positive, are dominated by repulsive interactions. Here we wish to make it explicitly clear that, for intermolecular separations greater than the equilibrium value (that is, before the energy ‘turn-around'), the magnitude of the *r*^6^ term is indeed larger in all cases, and dominates the *r*^12^ term, but that contribution of the *r*^6^ term is very similar for all the orientations.

It must be noted, however, that the interplay between the two terms is somewhat subtle. If we examine the ratio of the differences (Fig. 4d), then it is clear that the *r*^6^ term does begin to dominate the difference in the energies at around 1.06–1.1 nm separation. However, by reference to (Fig. 4c) it becomes clear that at this separation the difference in the potentials is <5 meV, that is, below our experimental sensitivity. Therefore, our modelling suggests that at the point at which the potential curves for different orientations become experimentally distinguishable, the difference between them is dominated by repulsive, rather than dispersive, interactions. As such it seems likely that although the magnitude of the variation in energy due to the variation in dispersion interaction under rotation of the molecule might in principle be within the noise limit of current DFM techniques, its direct measurement will always be hindered by the intrinsic convolution of the variation in energy due to repulsive forces, even at intermolecular separations significantly greater than the equilibrium value, where the gradient in the potential is positive.

We have presented 3D mapping of the variation in intermolecular interaction under changes in rotational orientation of a complex molecule with sub-Angstrom resolution via the functionalization of a scanning probe tip. Using a novel visualization method we can directly observe the variation in the value and position of the minimum of the potential energy as the orientation of the molecules is varied. By comparison of our results to both simple analytical and *ab initio* simulations, we are able to show that the variation in binding energy across the molecule is dominated by the onset of repulsive interactions between the front-most parts of the molecules. Surprisingly, we also find that variation in the net attractive part of the potential due to rotation of the molecules is still dominated by the repulsive forces, and the majority of the molecule only adds a uniform background to the potential. We anticipate that similar experimental techniques to those described here could be utilized to intuitively visualise the reactivity across complex interatomic and intermolecular potentials, including molecules with polarized or hydrogen bonding end groups.

## Methods

### Experimental methods

Clean Si(111)-7 × 7 surfaces were prepared by flash annealing a silicon wafer to 1,200 °C, rapid cooling to 900 °C and then slow cooling to room temperature. A low coverage of C_60_ was prepared by depositing the molecules from a home made tantalum pocket deposition source onto the room temperature substrate. Post-deposition, the sample was transferred into the scan head of an Omicron Nanotechnology LT DFM operating in UHV at cryogenic temperatures, and left to cool to 5 K before imaging.

Commercial qPlus sensors (Omicron Nanotechnology GmbH) with electrochemically etched tungsten wire tips were introduced into the scan head without any further preparation. The sensors were first prepared on clean Si(111)-7 × 7 surfaces by standard STM techniques until good STM/DFM resolution was achieved. Single C_60_ molecules were transferred to the tip by close approach to surface-adsorbed molecules, and the functionalization of the tip was checked by inverse imaging of the tip adsorbed molecule on the surface adatoms (Supplementary Figs 2,4 and 5)^7^. In all experiments an oscillation amplitude (A_0_) of 110pm was used, and the tip-sample bias was set to 0V. Three-dimensional Δ*f* volumes over the molecules were collected via the ‘slice' method^17^ and site specific (short-range) Δ*f* values were extracted using the ‘on–off' method^18^,^19^ then converted to potentials using the Sader–Jarvis algorithm^20^. Due to the long acquisition times required, residual thermal drift and piezoelectric creep were corrected using a custom atom-tracking and scripting setup^21^,^22^. Further details on the experimental setup, data processing steps and additional experimental data sets may be found in the Supplementary Methods.

### Flexible tip model and simulated spectroscopy procedure

To simulate DFM images, we adapted the method proposed by Hapala *et al*.^12^ to model the interaction between a sample and a CO-functionalized DFM tip. In our simulation the functionalized tip is assumed to consist of a tip base (outermost atom of the tip) and a probe. The probe is the flexible end of the model tip, and is allowed to move around the tip base. In our simulation, the probe is a C_60_ molecule consisting of 60 carbon atoms acting as a single effective probe particle attached to the tip base (Supplementary Fig. 6). Each atom in the probe experiences three forces; (i) a Lennard–Jones (L–J) force due to the tip base, (ii) a sum of all pairwise L-J forces due to interactions with atoms in the sample and (iii) a lateral harmonic force from the tip base. The net force on the probe is calculated by summing up all the forces experienced by each atom on the probe. The L–J interactions between atoms *α* and *β* are written as


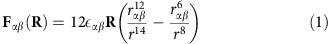



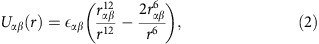


where *r*=|**R**| is the distance between atoms *α* and *β*, 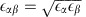 is the pair-binding energy and *r*_*αβ*_=*r*_*α*_+*r*_*β*_ is the equilibrium separation of the two atoms with 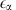 and *r*_*α*_ being the atomic parameters. In our calculations the L–J parameters for the carbon atoms were set to 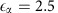 meV and *r*_*α*_=1.966 Å to ensure consistency with the work of Girifalco *et al*.^16^ and our own earlier work^7^. For the tip base a value of *r*_*α*_=5.0 Å was chosen, in order to take into account the larger size of the C_60_ molecule. The probe lateral stiffness and apex L–J parameter were set to *k*_*xy*_=0.5 N m^−1^ and 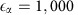 meV, respectively (Supplementary Figs 14–15). We acquired the simulation data by scanning the sample laterally with a step of Δ*x*, Δ*y*=0.1 Å. At each lateral position we placed the tip base at an initial separation *z*_0_=22 Å from the surface molecule and approached the sample (in our simulations another C_60_ molecule) in steps of Δ*z*=0.1 Å until *z*=17.5 Å allowing the probe position to be relaxed at each step due to the combined force of the sample and tip base. Note, however, that for ease of comparison to the DFT simulations all molecular separations discussed in the paper are given relative to the initial vertical core-core separation of the probe C_60_ from the surface C_60_.

### Density functional theory

DFT calculations were performed using the same initial high symmetry geometry (as described in main text) as the L–J simulations using the open source CP2K/Quickstep code^23^,^24^ utilising a hybrid Gaussian and plane-wave method^25^. Goedecker, Teter and Hutter pseudopotentials^26^ and the Perdew Burke Ernzerhof generalized gradient approximation method^27^ were used with a 300 Ry plane-wave energy cutoff. To account for dispersion interactions we employed the Grimme DFT-D3 method^28^, which well reproduced the C_60_–C_60_ pair potential (Supplementary Fig. 19) A double-zeta Gaussian basis set plus polarization (DZVP-MOLOPT)^29^ was used with a force convergence criterion for geometry relaxation of 0.05 eV Å^−1^. Geometry relaxation was carried out by allowing all atoms to relax other than the hexagonal faces of each molecule furthest apart from one another (to simulate attachment to the surface/tip).

## Additional information

**How to cite this article:** Sweetman, A. *et al*. Visualizing the orientational dependence of an intermolecular potential. *Nat. Commun.* 7:10621 doi: 10.1038/ncomms10621 (2016).

## Supplementary Material

Supplementary Information Supplementary Figures 1-22, Supplementary Methods and Supplementary References
