# Supplementary material for: Visualizing the orientational dependence of an intermolecular potential
Source: Nat Commun. 2016 Feb 16;7:10621. doi: 10.1038/ncomms10621 (PMC4757755; doi:10.1038/ncomms10621)
Supplement: Supplementary Information — Supplementary Figures 1-22, Supplementary Methods and Supplementary References [file ncomms10621-s1.pdf]

SUPPLEMENTARY FIGURES

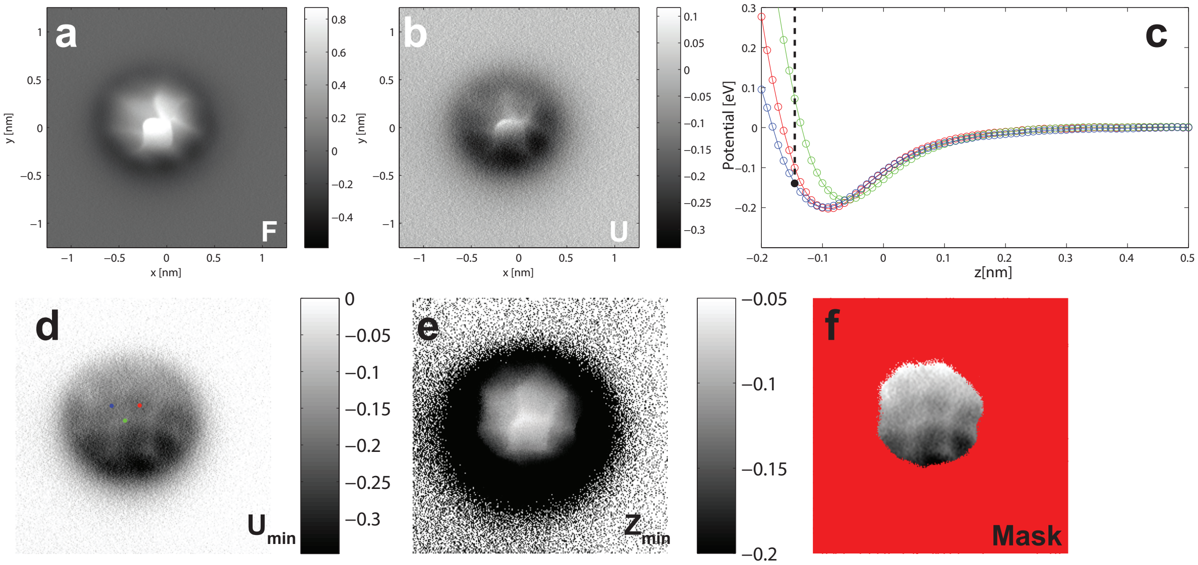

Supplementary Figure 1. Experimental measurement of variation in potential for a 3rd C_{60} molecule taken with the same tip as the dataset shown in Fig. 2 of main paper. Constant height images of a) Force (in nN) and b) Energy (in eV). c) Representative $U(z)$ curves taken at different positions across the left hand C_{60} molecule, dotted line shows the height of the force and energy slices shown in a) and b). d) Image showing the variation in the value of the energy (in eV) at the minimum in the $U(z)$ curve at each position in the 3D field. The positions of the curves shown in c) are marked. e) As for d), but showing instead the z height (in nm) at which the minimum occurs. Note that the black regions indicate regions where the minimum is found at the lowest tip-sample separation (i.e. no turnaround detected). f) Variation in energy minimum masked using the minimum in z position, red colouring indicates where the minimum in $U(z)$ is not present in the dataset.

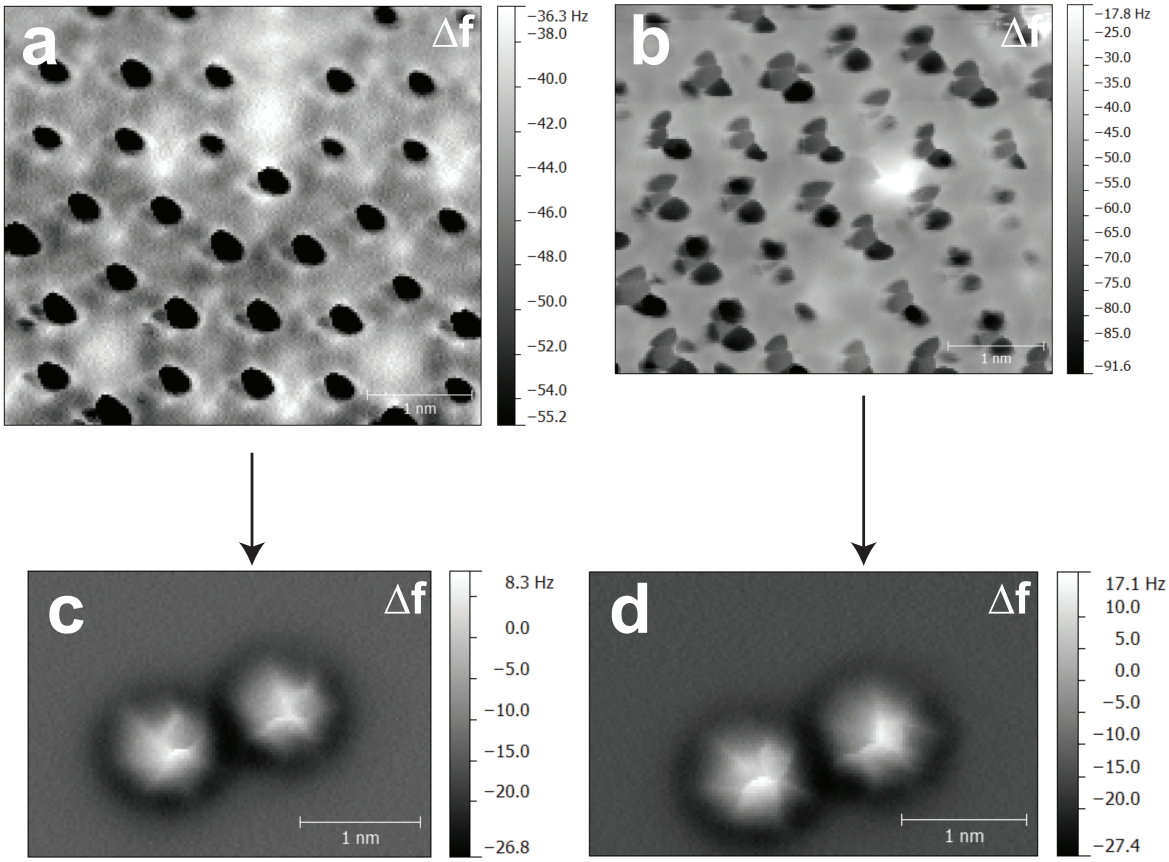

Supplementary Figure 2. Inverse imaging of the tip state on the Si(111)-7x7 adatoms. a) Initial orientation of tip-adsorbed C_{60} and c) corresponding contrast during imaging of surface adsorbed C_{60} with the same tip state. b) Orientation of tip-adsorbed C_{60} after rotation of molecule d) corresponding contrast during imaging of the same surface adsorbed C_{60} with the new tip state.

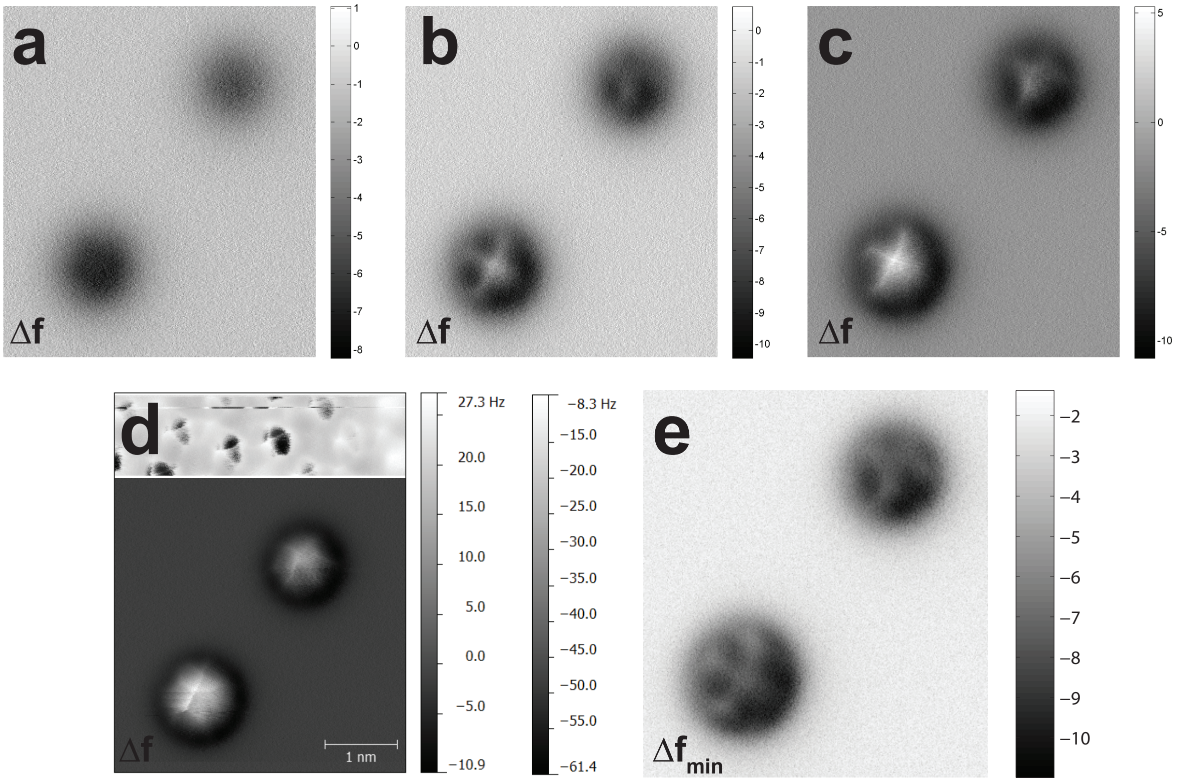

Supplementary Figure 3. Additional dataset showing evolution of Δf (in Hz) contrast during imaging of another set of surface adsorbed C_{60} with a different C_{60} terminated tip. a) $z =$ -0.18nm, b) $z =$ -0.26nm, c) $z =$ -0.30nm. d) Inverse imaging of tip state (top of image) and corresponding contrast over C_{60} (bottom of image). e) Δf_{min} image extracted from same dataset.

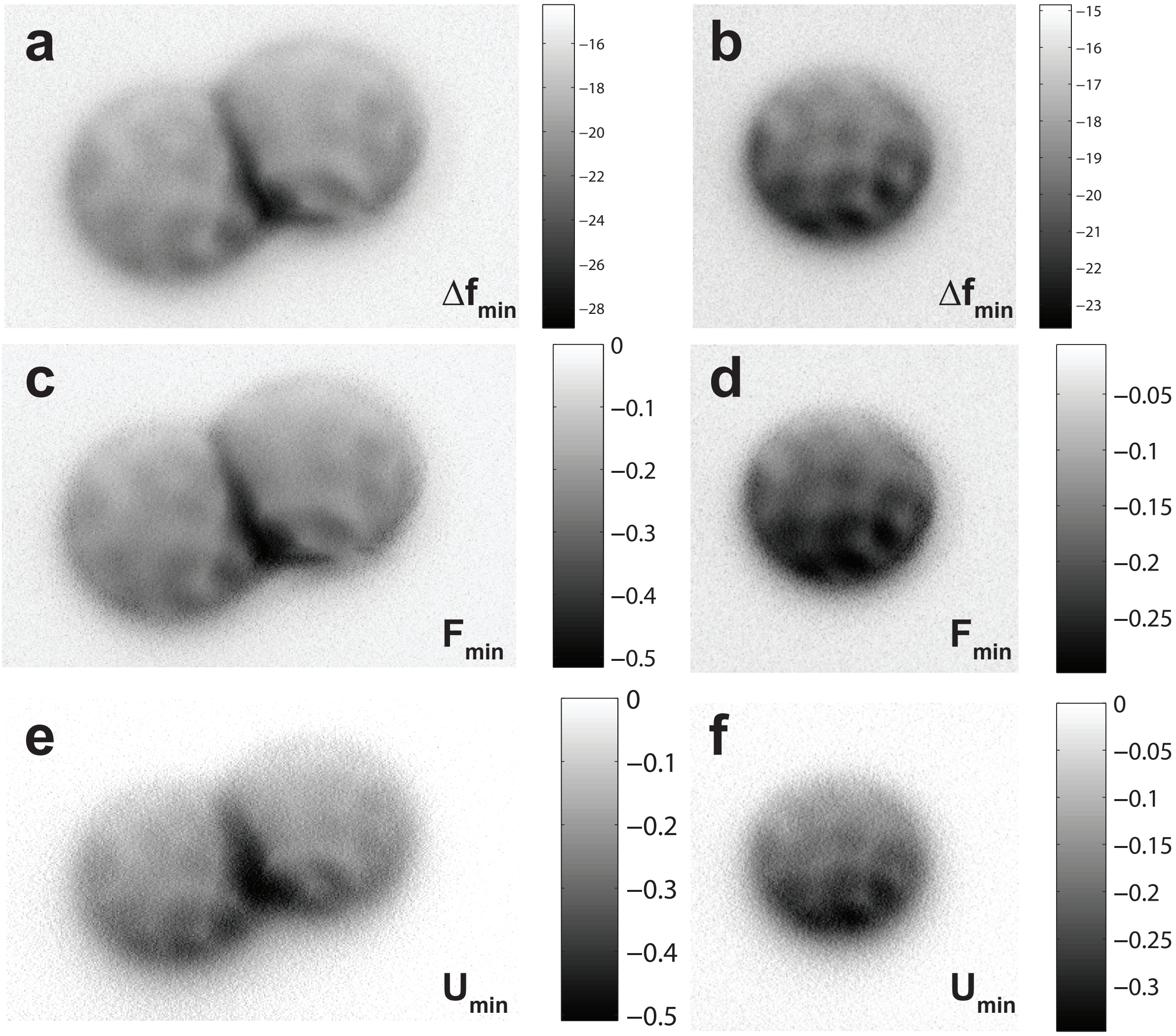

Supplementary Figure 4. a), b) Δf_{min} (in Hz). c), d) F_{min} (in nN), and e), f) U_{min} (in eV) images for 3 separate C_{60} molecules. Note that e) is the same image as shown in Figure 2 of the main paper.

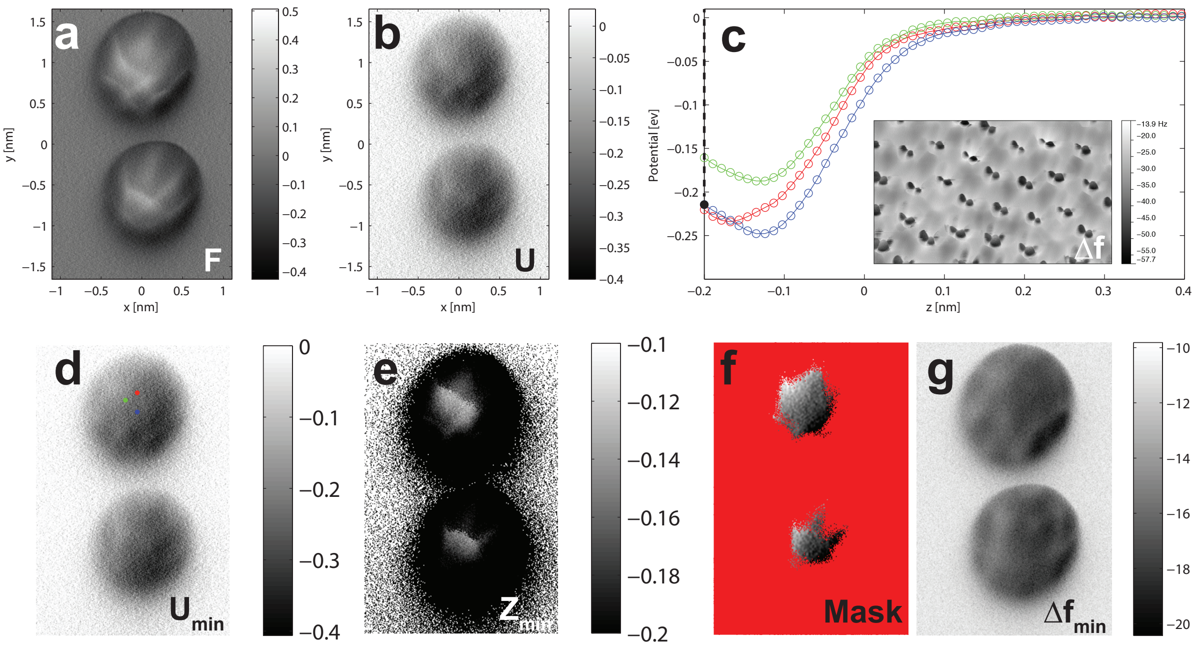

Supplementary Figure 5. Experimental dataset for C_{60} terminated tip demonstrating anomalous behaviour in the repulsive regime. Constant height images of a) Force (in nN) and b) Energy (in eV). c) Representative $U(z)$ curves taken at different positions across the left hand C_{60} molecule, dotted line shows the height of the force and energy slices shown in a) and b) - Inset constant height image showing inverse imaging of tip state on silicon adatoms. d) Image showing the variation in the value of the energy at the minimum in the $U(z)$ curve (in eV) at each position in the 3D field. The positions of the curves shown in c) are marked. e) As for d), but showing instead the z height (in nm) at which the minimum occurs, note that the black regions indicate regions where the minimum is found at the lowest tip-sample separation (i.e. no turnaround detected). f) Variation in energy minimum masked using the minimum in z position, red colouring indicates locations where the minimum in the intermolecular potential is not present in the $U(z)$ curve. G) Δf_{min} (in Hz) image extracted from same dataset.

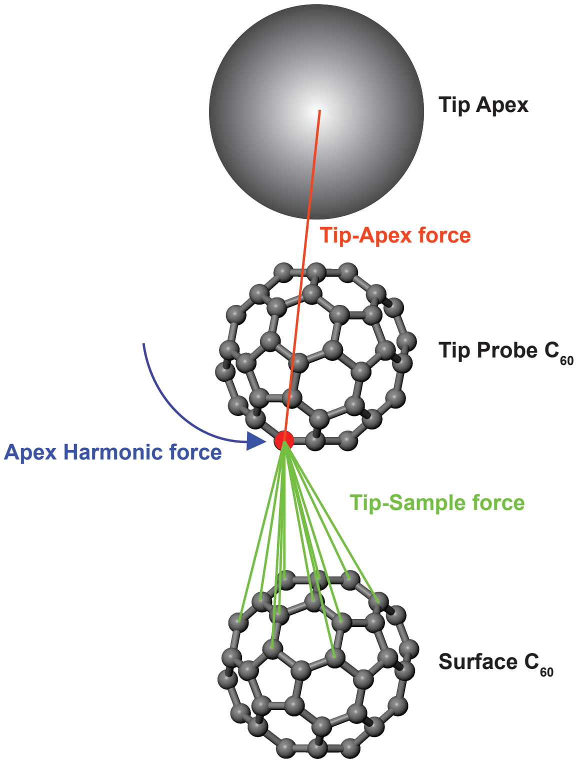

Supplementary Figure 6. Cartoon showing forces acting on each atom of the probe C_{60} molecule in the L-J model, after Fig. 1 of Hapala et. al. [1]

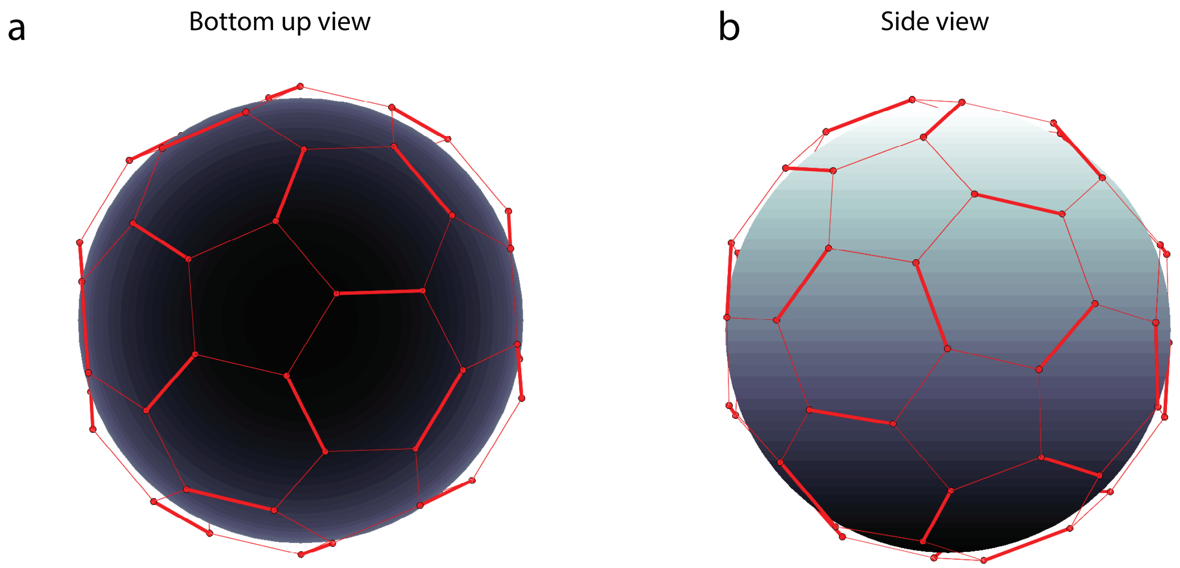

Supplementary Figure 7. a) Bottom up view (i.e. view from the surface molecule position) of the tip C_{60}, orientated with a tilted pentagon down. b) Side view of probe C_{60}.

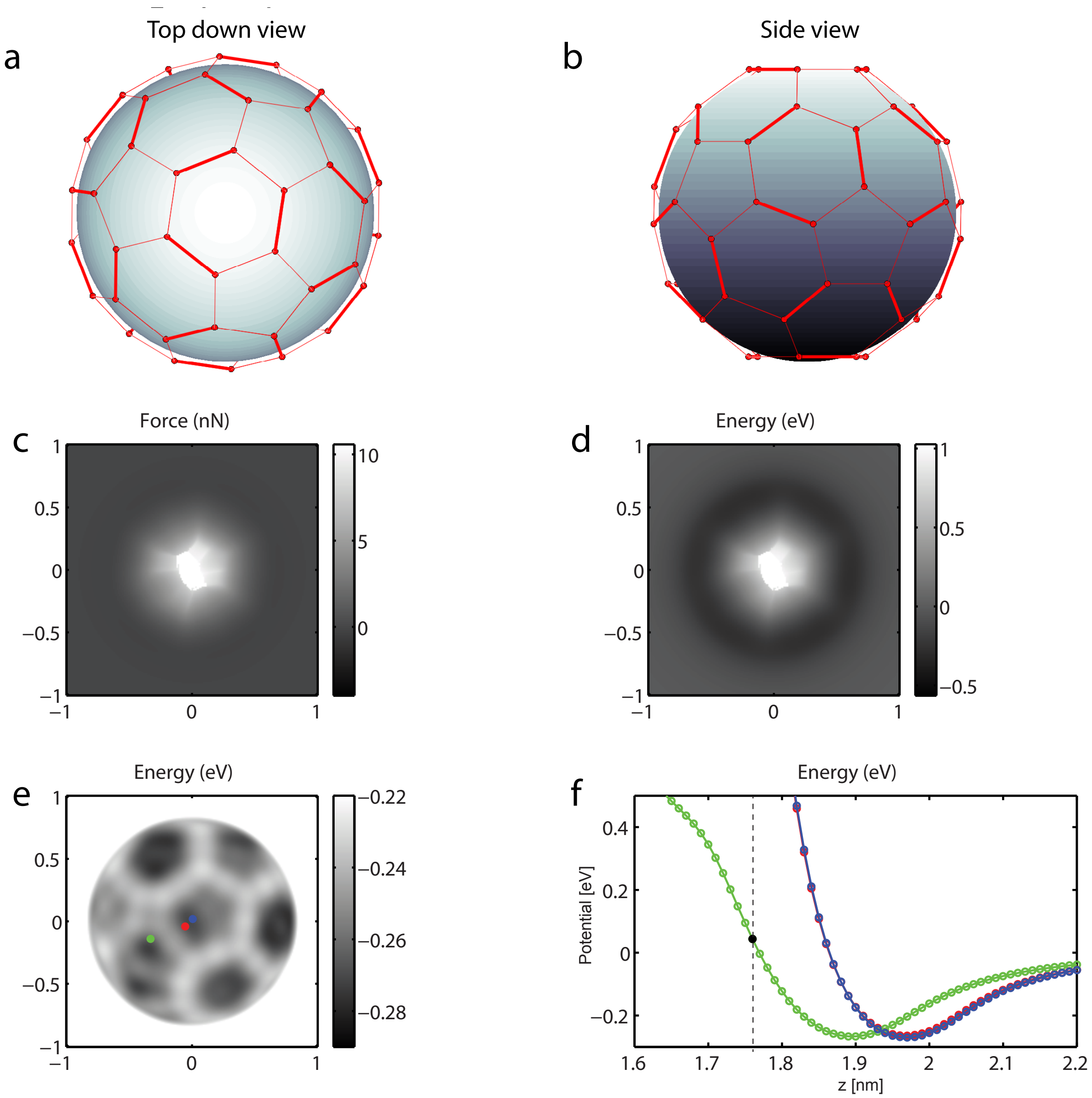

Supplementary Figure 8. a) Top down view (i.e. view from the tip molecule position) of the surface C_{60}. b) Side view of surface C_{60} molecule c) Simulated constant height force image (in nN) at close approach. d) Simulated constant height energy image (in eV) at close approach e) Simulated U_{min} image. f) Representative $U(z)$ curves taken at the positions indicated in e), black dot indicates position of constant height images.

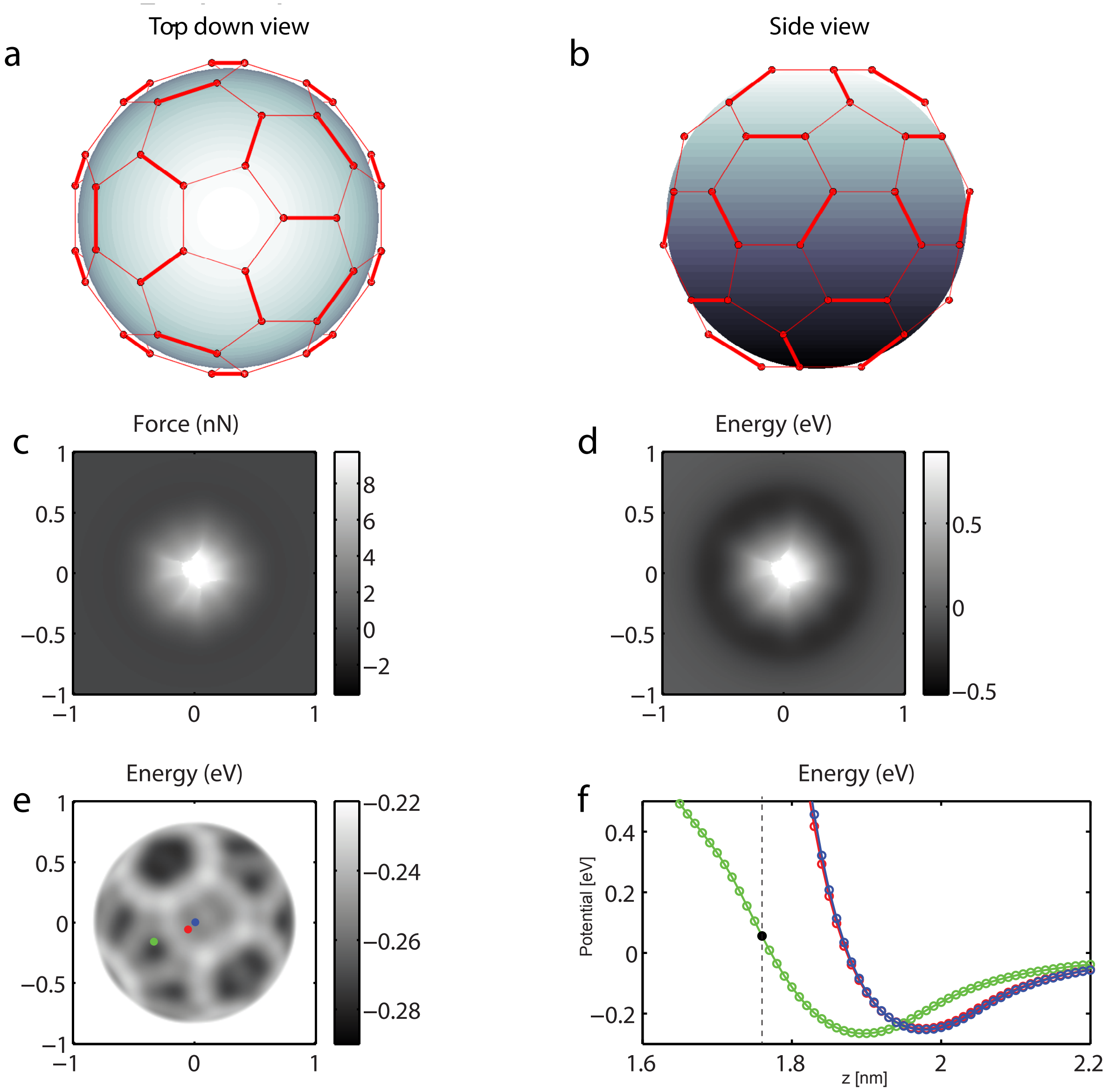

Supplementary Figure 9. a) Top down view (i.e. view from the tip molecule position) of the surface C_{60}. b) Side view of surface C_{60} molecule c) Simulated constant height force image (in nN) at close approach. d) Simulated constant height energy image (in eV) at close approach e) Simulated U_{min} image. f) Representative $U(z)$ curves taken at the positions indicated in e), black dot indicates position of constant height images.

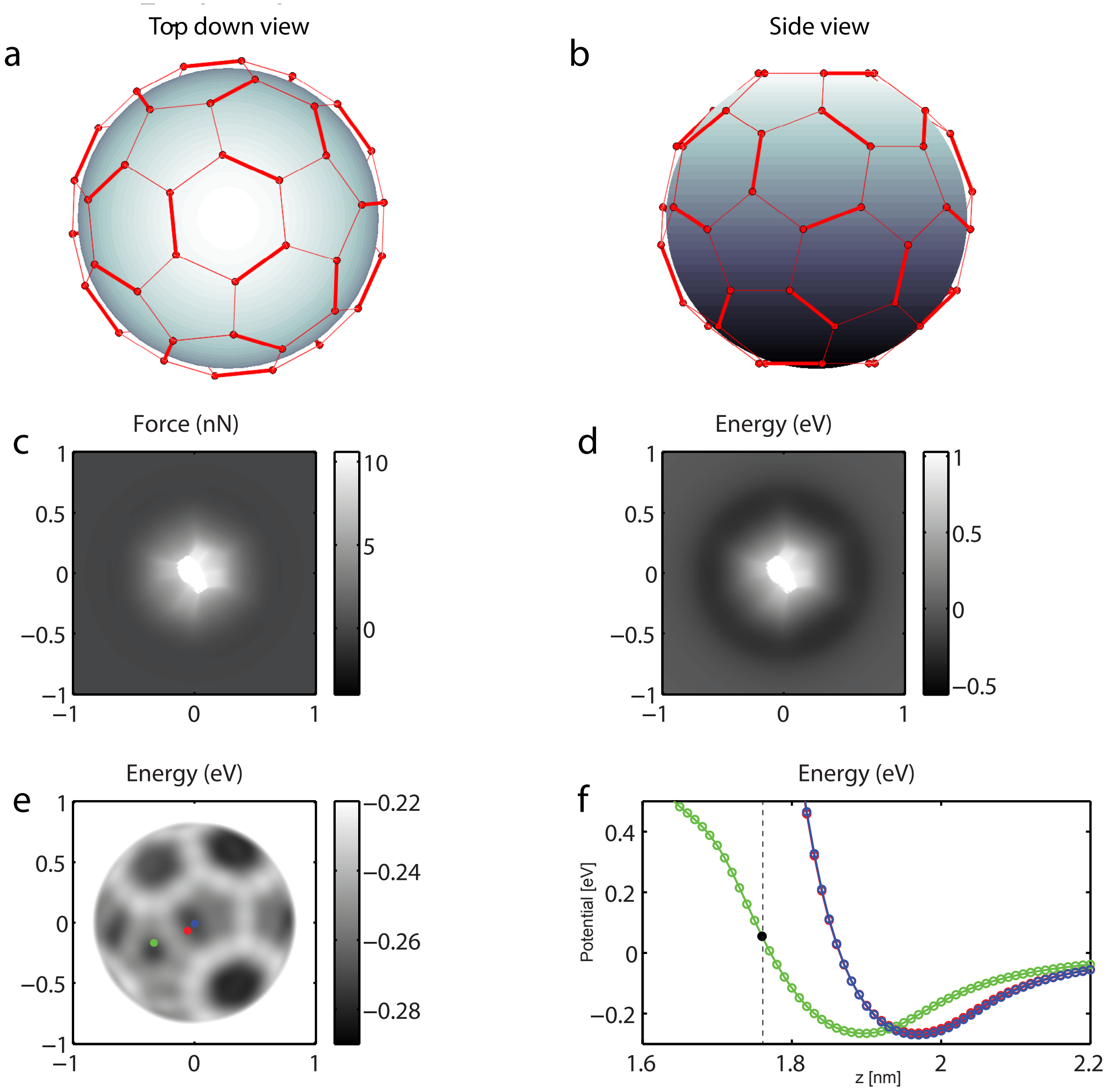

Supplementary Figure 10. a) Top down view (i.e. view from the tip molecule position) of the surface C_{60}. b) Side view of surface C_{60} molecule c) Simulated constant height force image (in nN) at close approach. d) Simulated constant height energy image (in eV) at close approach e) Simulated U_{min} image. f) Representative $U(z)$ curves taken at the positions indicated in e), black dot indicates position of constant height images.

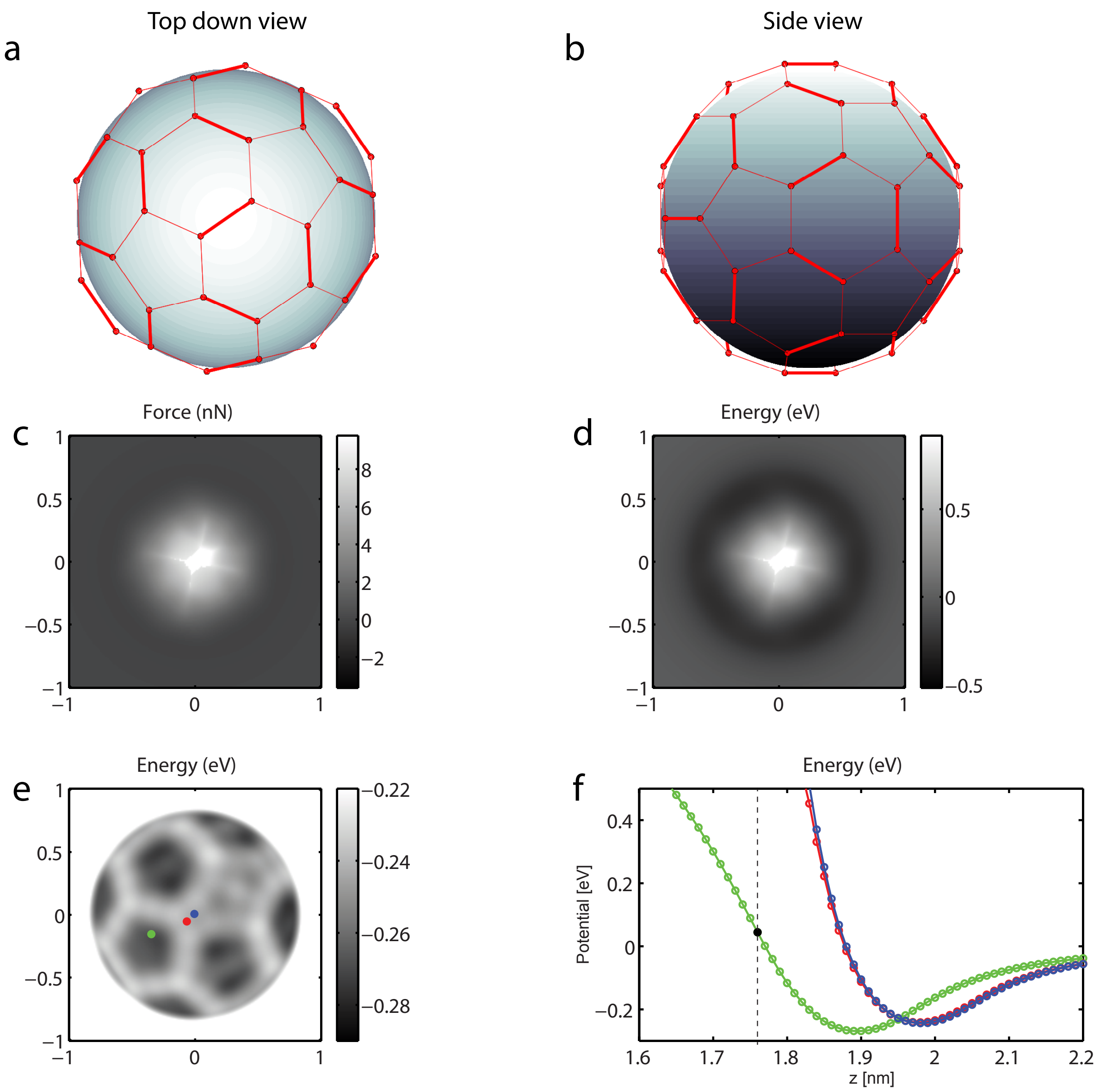

Supplementary Figure 11. a) Top down view (i.e. view from the tip molecule position) of the surface C_{60}. b) Side view of surface C_{60} molecule c) Simulated constant height force image (in nN) at close approach. d) Simulated constant height energy image (in eV) at close approach e) Simulated U_{min} image. f) Representative $U(z)$ curves taken at the positions indicated in e), black dot indicates position of constant height images.

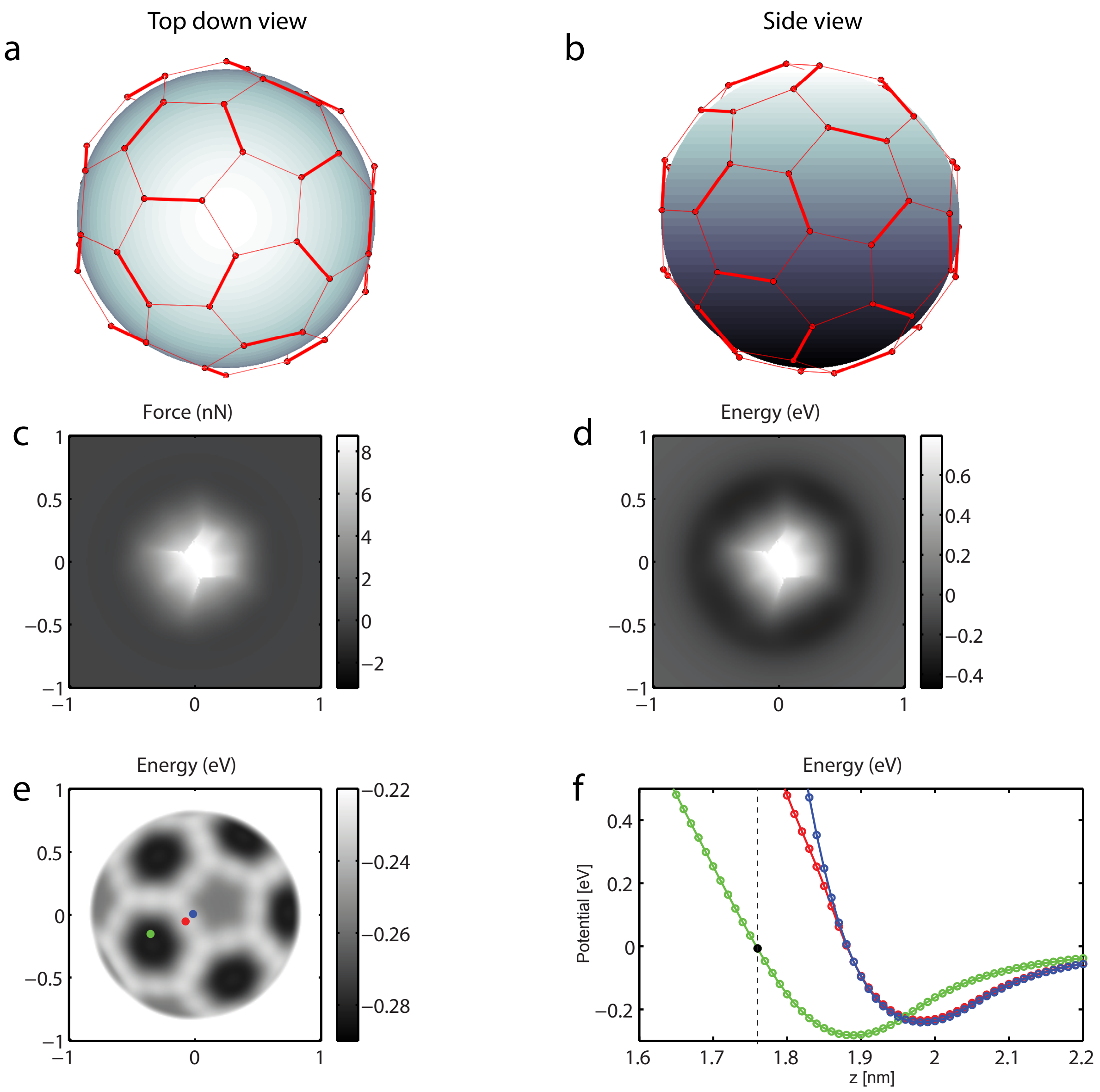

Supplementary Figure 12. a) Top down view (i.e. view from the tip molecule position) of the surface C_{60}. b) Side view of surface C_{60} molecule c) Simulated constant height force image (in nN) at close approach. d) Simulated constant height energy image (in eV) at close approach e) Simulated U_{min} image. f) Representative $U(z)$ curves taken at the positions indicated in e), black dot indicates position of constant height images.

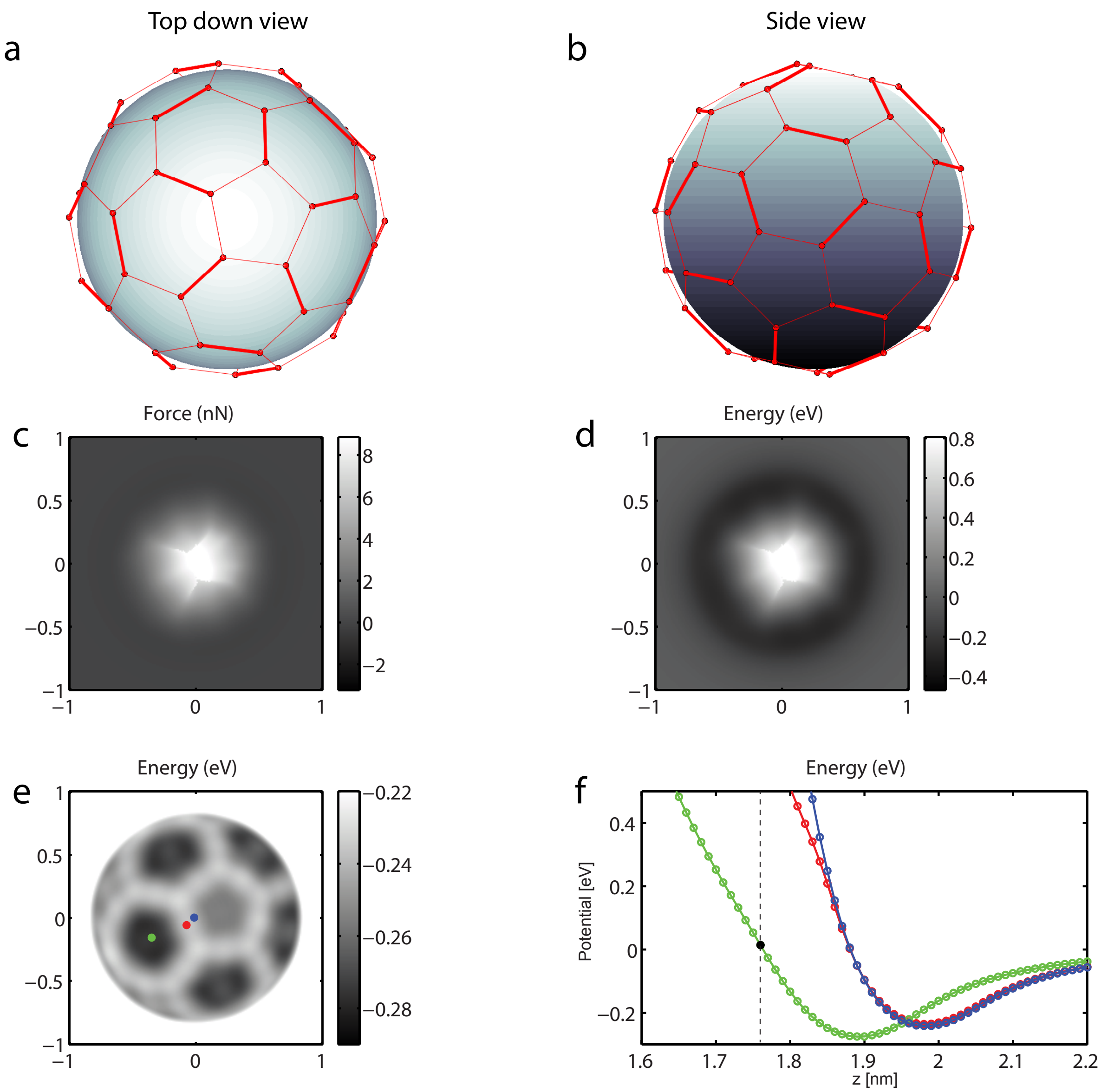

Supplementary Figure 13. a) Top down view (i.e. view from the tip molecule position) of the surface C_{60}. b) Side view of surface C_{60} molecule c) Simulated constant height force image (in nN) at close approach. d) Simulated constant height energy image (in eV) at close approach e) Simulated U_{min} image. f) Representative $U(z)$ curves taken at the positions indicated in e), black dot indicates position of constant height images.

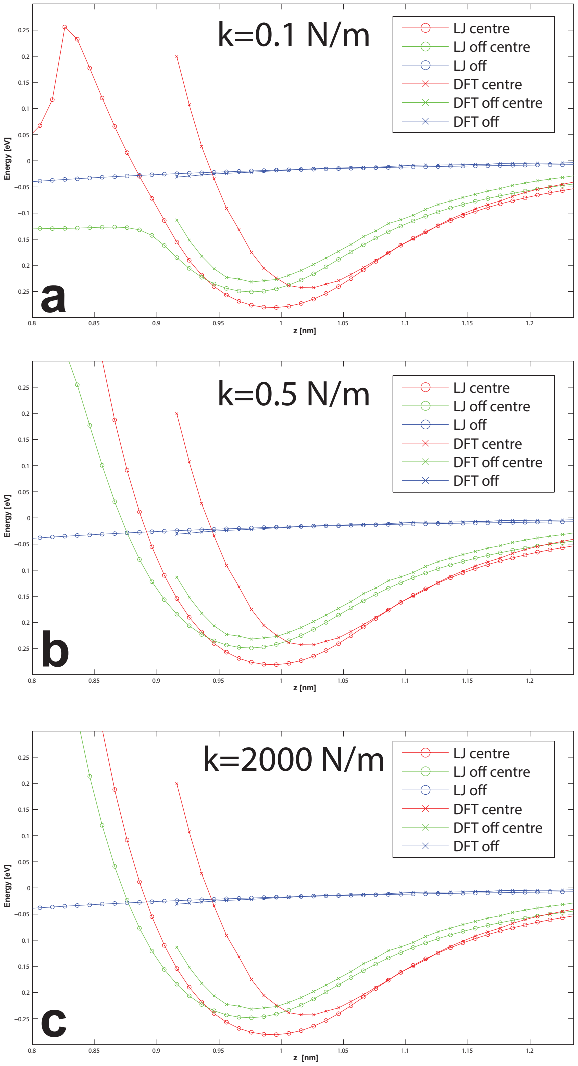

Supplementary Figure 14. Plots of $U(z)$ showing variation in energy curves as the lateral stiffness of the probe C_{60} is varied, compared to DFT simulation. a) k_{xy}=0.1 N/m, b) k_{xy}=0.5 N/m, c) k_{xy}=2000 N/m. Note that significant variation only occurs well into the repulsive branch of the potential.

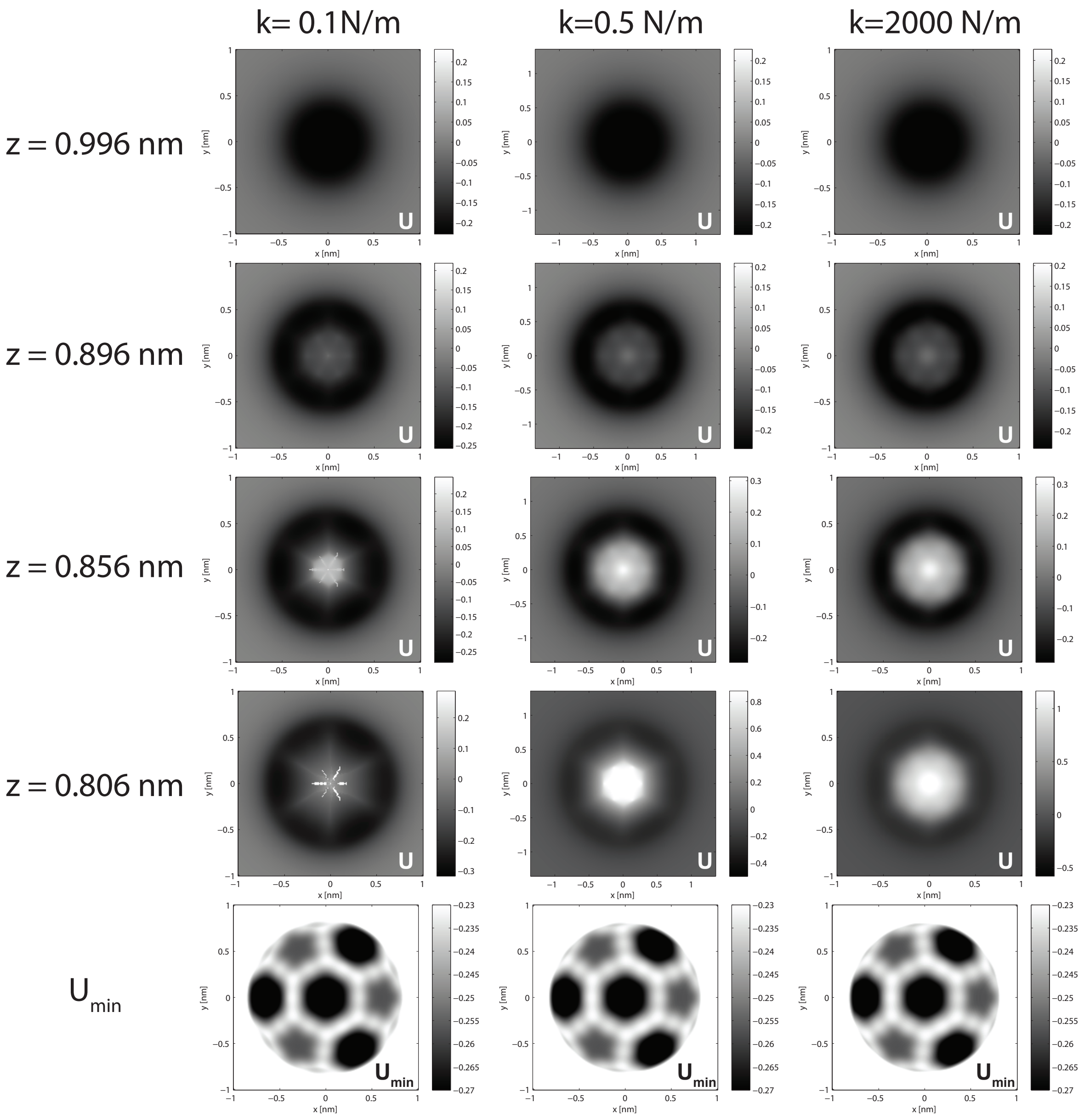

Supplementary Figure 15. Constant height energy images and U_{min} images (in eV) showing changes in imaging due to variation in lateral stiffness of probe C_{60}. Bright pixels at small intermolecular separation for k_{xy}=0.1 N/m simulations are due to failure of the simulation to converge in some positions at very close approach. Note that U_{min} images show almost no variation under changes in lateral stiffness.

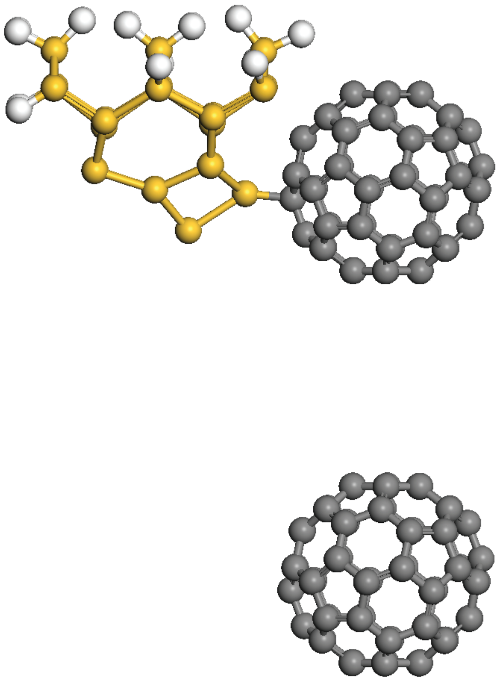

Supplementary Figure 16. Ball and stick model showing side view of geometry used in simulations testing the effect of backbonding. Silicon atoms are coloured yellow, hydrogen atoms are coloured white, carbon atoms are coloured grey.

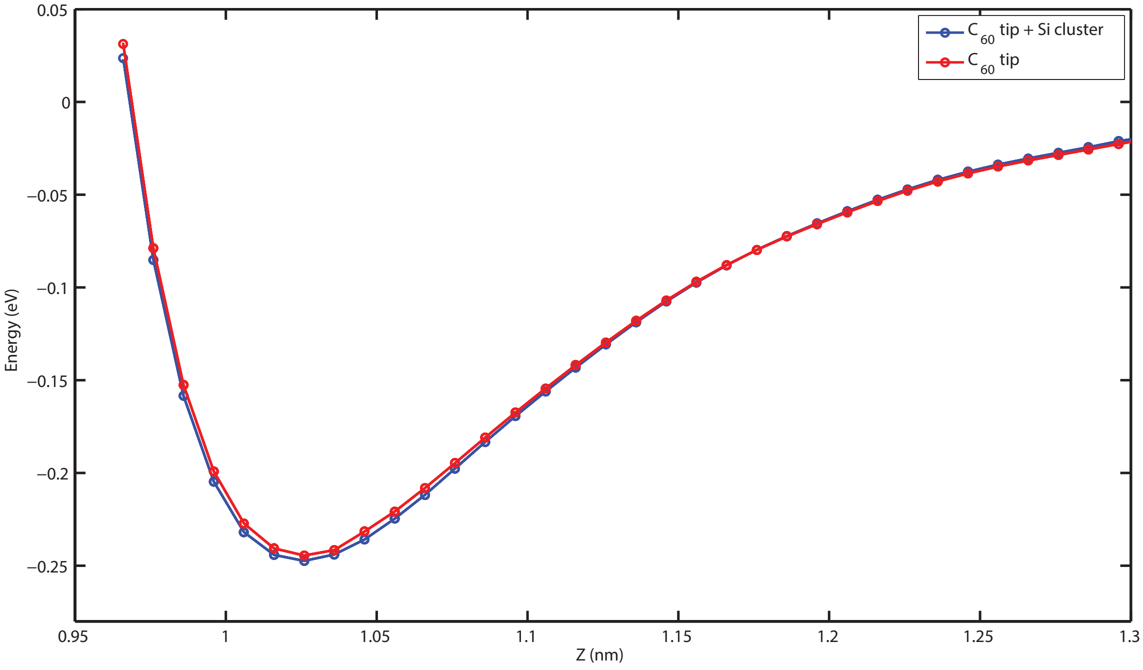

Supplementary Figure 17. $U(z)$ curves, calculated using DFT, showing interaction between the C_{60} + Si cluster tip, and bare C_{60} tip, and the surface C_{60}. The tip C_{60} was positioned directly over the centre of the surface C_{60} molecule in both cases.

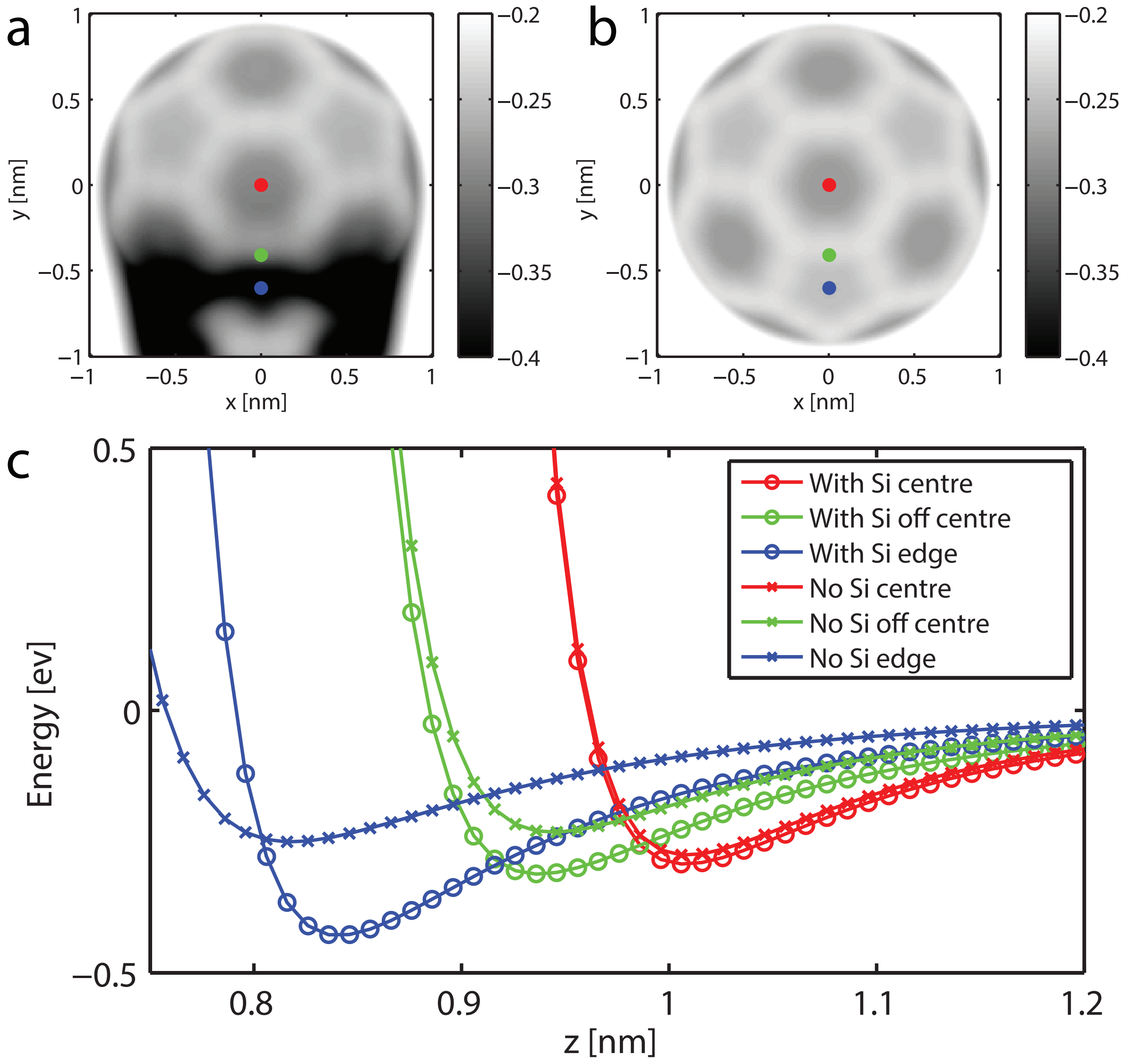

Supplementary Figure 18. a) U_{min} map calculated using L-J model for C_{60} + Si cluster tip. The influence of the Si cluster can be seen in the lower half of the image. b) U_{min} map calculated using L-J model for bare C_{60} tip. c) Representative $U(z)$ curves, taken at the positions indicated in a) and b). Over the centre of the molecule the effect of the Si cluster is relatively minor, but becomes significant in the locations where the cluster can interact strongly with the surface C_{60}.

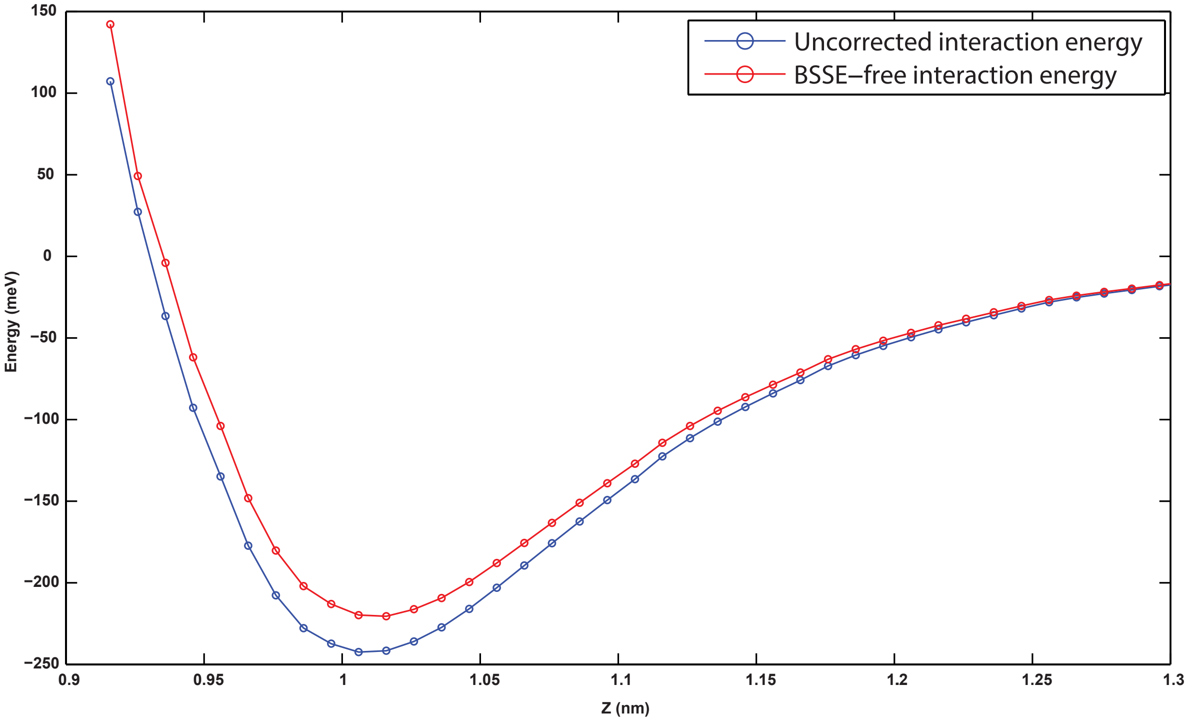

Supplementary Figure 19. $U(z)$ curves for the interaction between two C_{60} molecules with in the 'Hex - Hex' configuration, with, and without, correction for the BSSE.

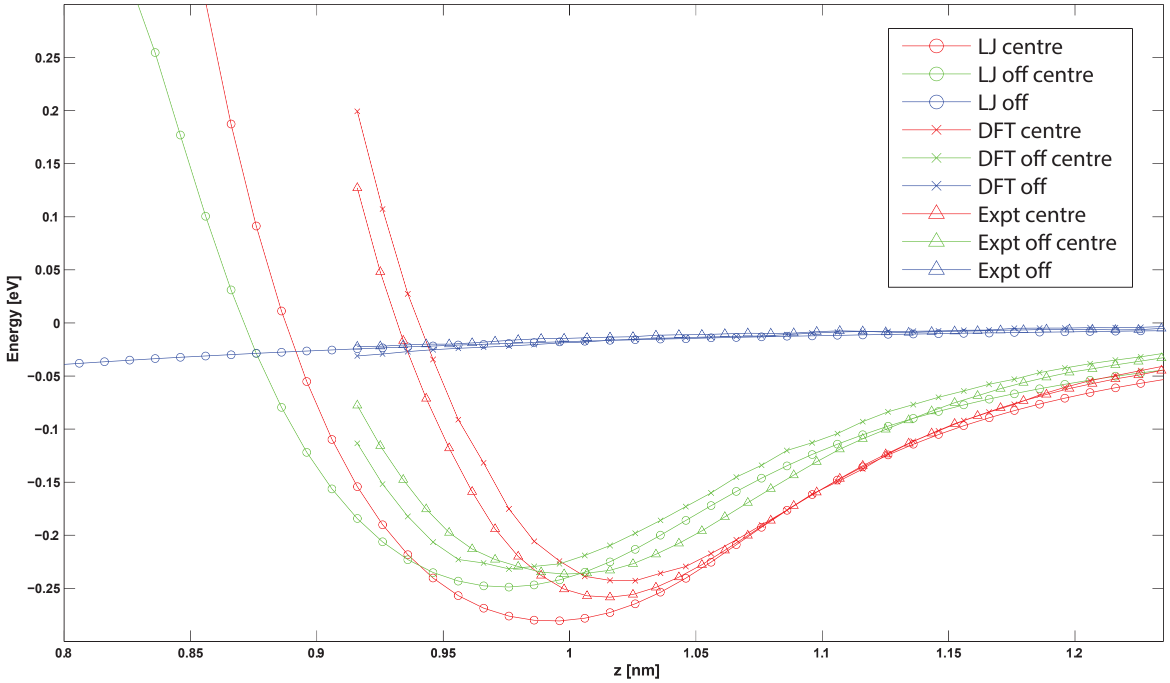

Supplementary Figure 20. $U(z)$ plots showing direct comparison of L-J simulation with k_{xy}=0.5 N/m , DFT simulation and experimental data. Note while the two simulations methods share the same absolute z axis, the z axis of the experimental dataset has been shifted to approximately align the minimum in the potential with the simulated data.

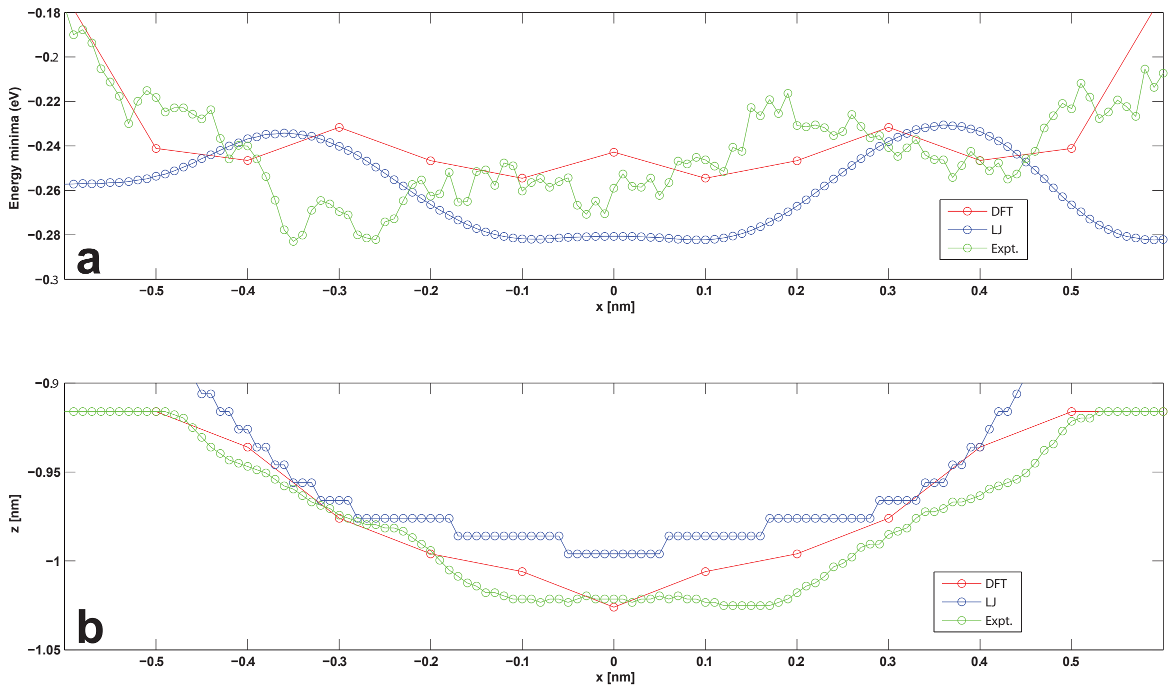

Supplementary Figure 21. Variation in a) U_{min} and b) z_{min} values with x displacement over the centre of the surface molecule for DFT simulation, L-J simulation with k_{xy}=0.5 N/m, and experimental data. The two simulation methods share the same absolute x axis. Note a simple smooth has been applied to the experimental plot to reduce the level of high frequency noise. The x axis of the experimental dataset has been shifted to approximately align the centre of the molecule with the simulated datasets

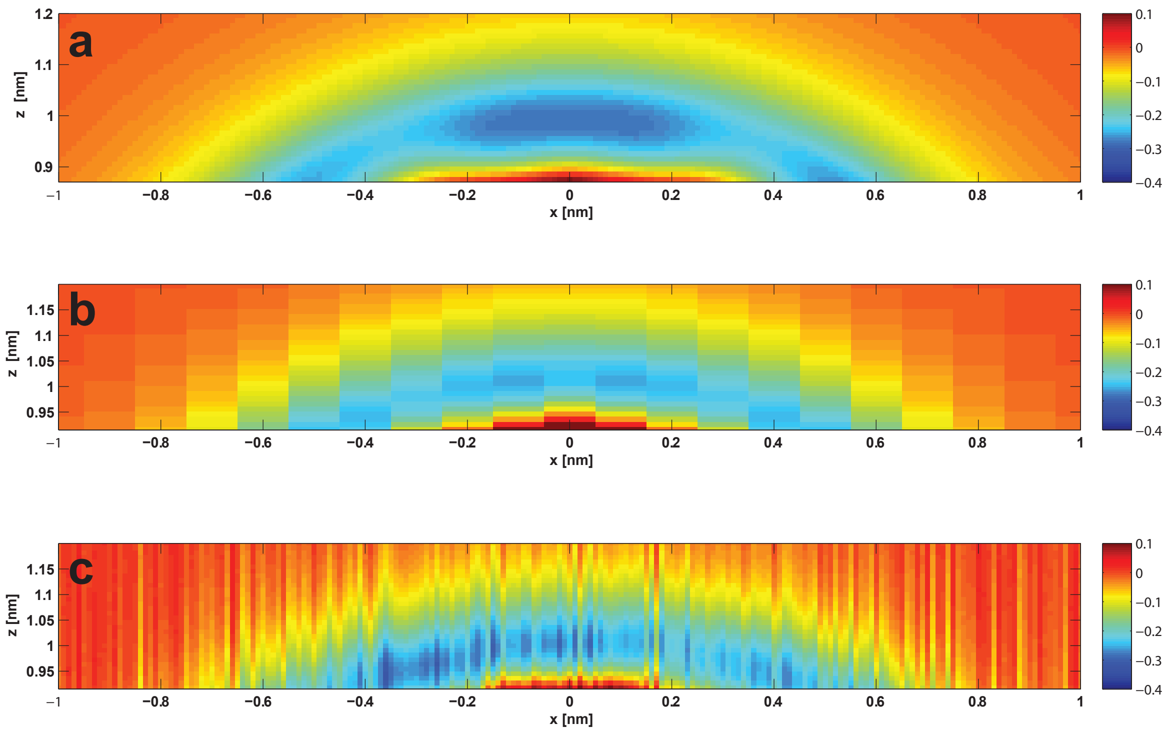

Supplementary Figure 22. xz plots of potential energy for a) L-J simulation with k_{xy}=0.5 N/m , b) DFT simulation and c) Experimental data (all in eV). The two simulations methods share the same absolute z and x axis. The z and x axis of the experimental dataset have been shifted to align the centre of the molecule and absolute z heights of the potential with the simulated data.

SUPPLEMENTARY METHODS

Processing of experimental data

As described in the main paper, 3D Δf datasets were acquired via the 'slice' method [2]. Typically, constant height images were acquired with a vertical spacing of 0.01 nm. Between each image the tip was placed over the molecule and an atom tracking protocol was used to stabilise the position of the tip and measure any change in the relative tip-sample position. Experimental z heights are given relative to the height in Δf feedback during atom tracking between images. Residual creep and thermal drift were compensated by application of a feed forward vector to the scan piezos. As a result of the acquisition protocol no post-processing of the images was required to align the dataset.

In order to create the 3D maps of potential we followed the following procedure. *U(z)* curves were extracted at a given position in the grid by subtracting an average 'off' $\Delta f(z)$ measurement (taken off the molecules) from the $\Delta f(z)$ curve at that point in the grid. This "on minus off" method (used for example by both Lantz et al, and Ternes et al [3, 4]) produced a "short-range" $\Delta f(z)$ curve that was then inverted to potential by the Sader-Jarvis method [5]. This procedure was repeated for each point in the grid to create the complete 3D volume. During inversion of the frequency shift data to potential via the Sader-Jarvis algorithm we used a representative tuning fork stiffness of k=2000 N/m. As discussed previously [6, 7] this value is likely subject to systematic errors of the order of +/- 10 %. As a result of the "on minus off" method the calculated force values only include site-specific interactions, as long-range van der Waals and electrostatic interactions are subtracted out. We explicitly note, however, that any site specific van der Waals interactions (that is to say, the short range dispersion attraction between the molecule on the tip and the molecule on the surface) are still included (and, as described below, are also accounted for in our density functional theory calculations). Force measurements were acquired by numerically differentiating the $U(z)$ curves using a Lanczos differentiator with a width set to 5 [8]. We also cross-checked the $U(z)$ and $F(z)$ curves extracted from the 3D datasets with $U(z)$ and $F(z)$ curves acquired by standard single point $\Delta f(z)$ spectroscopy measurements (with a higher pixel density in the z axis) and found them to agree within error. All xy maps of Δf, F, U, and U_{min} in the main paper are presented raw with no further processing. Single $U(z)$ and $F(z)$ curves extracted and plotted from 3D datasets are averages taken over a 3x3

pixel area.

Supporting datasets

Supplementary Figure 1 shows constant height force and energy images, plots of representative $U(z)$ curves, U_{min}, z_{min}, and masked U_{min} images for a 3rd C_{60} molecule mapped with the same tip as shown for Fig. 2 of the main paper.

In Supplementary Figure 2 we present scans showing inverse imaging of the tip state prior to acquisition of the 3D dataset shown in the main paper. Initially, the tip-adsorbed C_{60} was orientated in what appears to be an almost atom-down configuration, and after close approach to another molecule the tip molecule rotated to what appears to be a tilted hexagon or pentagon configuration. The corresponding contrast over the C_{60} molecules is shown below each image.

F_{min} and Δf_{min} images

Supplementary Figure 4 presents complimentary F_{min} and Δf_{min} images for the three molecules investigated in the main paper. The corresponding U_{min} images are also included to enable a direct comparison.

Reproducibility of Δf contrast

In Supplementary Figure 3 we show a series of constant height images, and corresponding Δf_{min} image, taken from another dataset using a C_{60} terminated tip, in order to show the reproducibility of the evolution in Δf contrast. We also show inverse imaging of the tip on the Si(111)-7x7 substrate in order to confirm the C_{60} termination of the tip.

Anomalous behaviour in repulsive regime

Although we generally find a high degree of reproducibility in the contrast and quantitative forces extracted for C_{60} on C_{60} measurements, we occasionally observe tips that exhibit anomalous behaviour. Supplementary Figure 5 shows an example of a dataset acquired with a tip of this type. The primary difference in these instances is the reduction in the gradient of the potential on the repulsive branch of the $U(z)$ curve, and the correspondingly more

'diffuse' appearance of the C_{60} in the F and U constant height images. Since we are able to identify the termination of the tip as being C_{60} via inverse imaging on the adatoms, this suggests that the deviations we observe are likely due to the stability of the adsorption state of the tip C_{60}. In particular, it is possible that in this instance the front most C_{60} is only weakly bound to the silicon tip, or possibly to a cluster of C_{60} molecules at the tip apex. We note that a similar reduction in the repulsive gradient of the $U(z)$ curves is observed in simulations with a very low lateral stiffness (See Supplementary Figure 15), although it is important to note that the diffusive nature of the constant height images is not reproduced, with the 'sharpening' instead becoming more intense for lower values of k_{xy}. Intriguingly, the quantitative appearance of the Δf_{min} image in this dataset is somewhat similar to those presented in Supplementary Figure 4, similar to the relative insensitivity of the U_{min} images in simulations carried out with high and low lateral stiffness. Consequently, this suggests that there may be additional complexity (for example additional degrees of freedom such as rolling, rotation, or cage distortion) that can also influence the behaviour of the tip adsorbed C_{60} that are that are not fully captured by the simple L-J empirical model.

SUPPLEMENTARY SIMULATION METHODS

Additional model data

Supplementary Figure 6 shows a schematic of the L-J model highlighting the decomposition of the forces that act on each carbon atom of the probe C_{60} molecule. The probe C_{60} is free to rotate around the apex particle, subject to a harmonic restoring force as described in the main paper. The apex-probe force keeps the probe molecule attached to the tip, but also allows for some variation in the probe-apex separation. Note that the probe C_{60} maintains a fixed orientation during rotation (i.e. all the atoms in the molecules undergo a uniform translation and therefore maintain the same orientation relative to the surface).

Simulations using realistic C_{60} - C_{60} orientations

In this section we show the results of L-J simulations performed with complex C_{60} - C_{60} orientations, that more closely mimic the orientations encountered experimentally, as compared to the high symmetry 'Hex-Hex' orientation shown in the main paper. Supplementary Figure 7 shows the tip orientation used throughout this series of simulations, which

is oriented with a tilted pentagon towards the surface in order to emulate the orientation observed during the inverse imaging of the tip molecule shown in Supplementary Figure 2 b). The sample C_{60} orientation was varied through a number of positions corresponding to known C_{60} adsorption geometries on Si(111)-7x7 [9]. These geometries, and the results of the simulations, are shown in Figs. S7 - Supplementary Figure 13.

In the experimental datsets presented in this paper we did not observe clear molecular orbital resolution during STM imaging at 5K, most likely because after extensive preparation on the silicon surface a large bandgap develops on our tip, and the conductivity is further reduced during operation at low temperature. As a result we were not able to uniquely determine the orientation of the surface adsorbed molecules investigated in the main paper via deconvolution of the molecular orbitals (as presented in Lakin et. al. [10] for example), as although the adsorption position within the unit cell could be determined, this is not sufficient to uniquely determine the rotational orientation relative to the tip [11]. Therefore, while these simulations provide an important, and more realistic, insight into the complex convolution effects resulting from the low symmetry orientations that C_{60} possesses on the Si(111)-7x7 surface, it is important to stress that the configurations simulated here do not necessarily represent the exact configurations probed experimentally. Nonetheless, the qualitative appearance of both the constant height slices and the complex patterns exhibited on the U_{min} images are closer to those observed experimentally than for the high symmetry case 'Hex-Hex' orientation, which is used in the main paper to make a direct comparison to the DFT simulations. We also note that the choice of orientation only causes small changes in the peak-to-peak variation in U_{min} image, due to the high number of effective orientations explored during 3D mapping of the potential over full range of initial x and y starting co-ordinates.

Effect of variation in k_{xy} on L-J simulations

In the flexible tip model proposed by Hapala et. al. it is necessary to set a number of empirically derived variables. One key parameter is the lateral stiffness of the probe particle. In the simulations shown in the main paper a stiffness of $k_{xy} = 0.5$ N/m is chosen, similar to that used in simulations of a CO tip. However, the lateral stiffness of the tip adsorbed C_{60} is not a well known quantity, and likely varies significantly with differences in bonding configuration. In this section we explore the effect on our simulated data due to varying the

lateral stiffness parameter to test the robustness of our conclusions.

In Supplementary Figure 14 and Supplementary Figure 15 we show the effect of varying k_{xy} on the $U(z)$ curves, and xy plots of U, respectively. We find that that the attractive part of the well and the value of the potential minimum are almost unaffected by the changes in k_{xy}, as the parameter only becomes critical when repulsive interactions between tip and sample begin to introduce large deflections in position of the probe particle. Similar to Hapala et. al. [1] for lower k_{xy} values we observe a reduction in the gradient in the repulsive regime, the 'sharpening' of the features becomes more prominent at larger separations, and we sometimes observe a 'turnover' in the $U(z)$ curve. However, we also observe some qualitative differences at lower stiffness's due to the more complex interaction between the two C_{60}'s (as compared to a simple point-like CO tip). Specifically for lower stiffness's we sometimes observe numerical convergence issues in the simulation that remain even when very high numerical tolerances and numbers of convergence steps are used. This can result in the tip C_{60} becoming artificially 'trapped' in certain positions over the surface C_{60}, and suggests a more sophisticated energy minimisation routine maybe required to take into account the more complex energy landscape for the C_{60} - C_{60} interaction in this regime. However, as we do not observe this behaviour in the majority of our datasets, and noting the relatively good agreement we observe between the experimental datasets, DFT and L-J simulation, we believe the choice of k_{xy}=0.5 N/m for the datasets presented in the main paper to be justified on empirical grounds.

Estimating the influence of tip backbonding

A key assumption made in measuring the variation in intermolecular binding energy by mounting a molecule on a scanning probe tip is that the backbonding of the molecule to the tip does not affect the value of the minimum in the potential. In particular, since in this study the extended dispersion interaction of the molecule is explicitly considered, some consideration must be given to the influence of the immediate part of the tip apex onto which C_{60} is mounted, as depending on the (experimentally unknown) geometry, some parts of the tip could in principle have a comparable influence on the surface C_{60} as parts of the tip C_{60}.

In our analysis of the data presented in Fig. 2 of the main paper, we note that we observe a gradient across the U_{min} image, which must result at least in part from the influence of the

tip structure, and therefore only consider variations in the potential measured over the centre of the molecule where the gradient is small. In this section we consider the effect of mounting a C_{60} molecule onto a small prototypical silicon tip cluster on the potential measurements and U_{min} image. We stress that these results are only intended to give a preliminary insight into the effect of the mounting of the molecule onto the tip, and a detailed consideration of the adsorption of C_{60} molecules onto large, realistic, silicon clusters would require an extensive DFT study, and is beyond the scope of this work.

Supplementary Figure 16 shows the mounting of a C_{60} molecule, with a hexagon face pointing down, on the silicon cluster, after relaxation of the geometry using DFT. The atomic positions were then fixed and used to perform simulated spectroscopy on a C_{60} with a hexagon face pointing up, either directly over the centre of the molecule (using DFT), or building up a complete 3D grid (using the L-J model). In the DFT simulation electronic relaxation was permitted, although the atomic positions were fixed. The same simulations were then repeated after removing the silicon cluster to provide a control measurement with the same atomic geometries. The choice to prevent geometric relaxation was made so as to isolate the influence of the vdW interaction from the silicon cluster from any modification to the tip flexibility caused by the mounting onto the cluster. This assumption was justified in the case of the L-J simulation by noting that allowing relaxation only has a small influence on the U_{min} image, as the minimum in the intermolecular potential usually occurs before significant deflection of the tip apex due to the lateral forces (see Supplementary Figure 15) .

The results of these simulations are shown in Supplementary Figure 17 and Supplementary Figure 18. The primary result is that the measurements performed over the centre of the molecule experience only demonstrate small shifts in the minimum due to the presence of the silicon cluster, with a difference in the minimum in the potential of 3 meV in the DFT and 17 meV in the L-J, compared to the simulations performed without the silicon cluster. In the simulated U_{min} map, performed using the L-J model, the extended influence of the silicon cluster can clearly been seen, resulting in a gradient across the U_{min} map on the lower part of the image. This clearly shows that the mounting of the molecule onto the cluster can affect the minimum in the potential at some positions, but we note that in these simulations the gradient does not extend across the entire molecule as is seen in some of our experimental datasets, most likely due to the small cluster size used in our simulations. In experiment it is likely that the molecule is mounted onto a much larger cluster, and it

seems possible (for example) that a large 'wedge' shaped cluster behind the molecule could generate a the gradient in U_{min} similar to that observed experimentally.

Estimating the basis set superposition error (BSSE)

In order to check the accuracy of our calculations, and in particular the suitability of using a DZP basis, we calculated the BSSE using the Boys-Bernardis counterpoise scheme as implemented in the CP2K code. These simulations showed that although the BSSE has an effect, it only serves to increase the calculated interaction energy by a maximum of 9%, reducing the value of U(z) at the energy turnaround from -281 meV to -258 meV for a pentagon-pentagon configuration and -242 meV to -220 meV for a hexagon-hexagon configuration. Supplementary Figure 19 shows the effect of the BSSE on the energy curves for simulated spectroscopy performed in the Hex-Hex configuration directly over the centre of the molecule.

Direct comparison of experimental and simulated data

Supplementary Figure 20 and Supplementary Figure 22 reproduce the combined plots of L-J and DFT energy from the main paper, with the addition of experimental data extracted from the single C_{60} dataset shown in Supplementary Figure 1 plotted on the same axis to enable direct comparison. As noted in the figure captions, while the two simulation methods share the same absolute coordinates, the absolute value of the x and z axis for the experimental data is arbitary. Therefore the x and z axis of the experimental data have been uniformly shifted to align with the simulated datasets. As such, while the width, depth and shape of the potentials can be directly compared, the position of the minimum is arbitrary for the experimental data. It is also important to note that as described in the main paper, the experimental orientation of the C_{60} molecules is not the same as the high symmetry Hex-Hex orientation used in these simulations, therefore the comparisons should be taken as representative only.

Supplementary Figure 21 shows the variation in U_{min}, and z_{min}, plotted at different x displacements across the molecule for the two simulation methods, and the experimental data shown in Supplementary Figure 20. While there is good agreement in the variation in z_{min} between the three plots the variation in U_{min} estimated from the DFT simulations is

smaller than that observed experimentally, or estimated from the L-J simulation.

SUPPLEMENTARY REFERENCES

---

[1] Hapala, P. *et al.* Mechanism of high-resolution STM/AFM imaging with functionalized tips. *Physical Review B* **90**, 085421 (2014).

[2] Neu, M. *et al.* Image correction for atomic force microscopy images with functionalized tips. *Physical Review B* **89**, 205407 (2014).

[3] Lantz, M. A. *et al.* Quantitative Measurement of Short-Range Chemical Bonding Forces. *Science* **291**, 2580–2583 (2001).

[4] Ternes, M. *et al.* Interplay of conductance, force, and structural change in metallic point contacts. *PRL* **106**, 016802 (2011).

[5] Sader, J. E. & Jarvis, S. P. Accurate formulas for interaction force and energy in frequency modulation force spectroscopy. *APL* **84**, 1801 (2004).

[6] Sweetman, A. *et al.* Toggling bistable atoms via mechanical switching of bond angle. *PRL* **106**, 136101 (2011).

[7] Chiutu, C. *et al.* Precise Orientation of a Single C_ {60} Molecule on the Tip of a Scanning Probe Microscope. *Physical review letters* **108**, 268302 (2012).

[8] Sweetman, A. & Stannard, A. Uncertainties in forces extracted from non-contact atomic force microscopy measurements by fitting of long-range background forces. *Beilstein journal of nanotechnology* **5**, 386–93 (2014).

[9] Rurali, R., Cuadrado, R. & Cerdá, J. I. C60 adsorption on the Si(111)-p(7x7) surface: A theoretical study. *Physical Review B* **81**, 075419 (2010).

[10] Lakin, A. J., Chiutu, C., Sweetman, A. M., Moriarty, P. & Dunn, J. L. Recovering molecular orientation from convoluted orbitals. *Physical Review B* **88**, 035447 (2013).

[11] Liu, L. *et al.* Switching molecular orientation of individual fullerene at room temperature. *Scientific reports* **3**, 3062 (2013).
